# A chicken lncRNA is identified as a critical regulator that increases influenza virus replication by impairing innate antiviral responses

**DOI:** 10.1186/s13567-025-01635-4

**Published:** 2025-10-15

**Authors:** Nelam Sajjad, Mohamed Maarouf, Yiming Wang, Prasha Shrestha, Kul Raj Rai, Xiaojuan Chi, Song Wang, Ji-Long Chen

**Affiliations:** 1 Fujian Agriculture and Forestry University, Fuzhou, China; 2 Key Laboratory of Animal Pathogen Infection and Immunology of Fujian Province, College of Animal Sciences, Fujian Agriculture and Forestry University, Fuzhou, China; 3 Key Laboratory of Fujian-Taiwan Animal Pathogen Biology, College of Animal Sciences, Fujian Agriculture and Forestry University, Fuzhou, 350002 China

**Keywords:** Chicken, H9N2 influenza virus, LncRNA, NF-κB, IL-6, interferon

## Abstract

Influenza A virus (IAV) poses a significant global health threat to both humans and animals. Increasing evidence highlights long noncoding RNAs (lncRNAs) as critical regulators of various physiological and pathological processes, but their roles in virus‒host interactions in chickens remain elusive. This study identified chicken lncRNAs and further investigated their functional involvement in IAV–host interactions. Transcriptome sequencing of chicken DF-1 cells revealed that 2118 lncRNAs were differentially expressed following H9N2 avian influenza virus infection. Among these, a lncRNA that we named lncRNA-up4 was significantly upregulated by H9N2 infection. LncRNA-up4 was predominantly localized in the nucleus but also detected in the cytoplasm. Furthermore, we found that the virus-induced expression of lncRNA-up4 was regulated by the pattern recognition receptor-dependent NF-κB signalling pathway. Functional analysis demonstrated that silencing lncRNA-up4 impaired IAV replication by upregulating the expression of several critical antiviral molecules, including IFN-β, MX1, and OAS-1. Conversely, overexpression of lncRNA-up4 increased viral replication, as evidenced by increased viral NP protein and virus titers. Moreover, we observed that lncRNA-up4 positively regulated the expression of IL-6, TNF-α, and IL-1β but suppressed the expression of IFN-β at the mRNA level. These results reveal that the newly identified lncRNA-up4 plays an important role in influenza virus replication through the regulation of cytokine production and antiviral gene expression in chicken cells. This study provides valuable insight into the regulatory function of chicken lncRNAs in innate immunity.

**Supplementary Information:**

The online version contains supplementary material available at 10.1186/s13567-025-01635-4.

## Introduction

Influenza A virus (IAV), an RNA virus of the *Orthomyxoviridae* family, is primarily an airborne pathogen that spreads through respiratory droplets and causes disease in humans and animals. IAV is notable for its high mutation rate and genetic reassortment ability, which facilitate the emergence of new strains and occasional pandemics [1, 2]. Avian influenza virus (AIV) is classified into two groups according to pathogenicity: highly pathogenic avian influenza virus (HPAIV) and low pathogenic avian influenza virus (LPAIV) [3]. HPAIV often results in high mortality rates in chickens and spreads to various tissues and organs, affecting the cardiovascular, respiratory, urinary, and nervous systems [4, 5]. Infections with LPAIV typically result in mild respiratory signs or are asymptomatic [6]. H9N2 is a LPAIV that poses a significant and ongoing threat to the global poultry industry [7]. Multiple hosts are susceptible to H9N2 infection, including avian and mammalian species [8]. Moreover, genetic reassortment between avian and other animals or human influenza viruses may produce new mutants with distinct traits that pose a risk to human and animal health [9]. For example, H9N2 is a potential contributor to this process and the emergence of highly virulent and transmissible AIV variants in humans and animals. Notably, the zoonotic H7N9 and H10N8 strains acquired internal gene segments (PB2, PB1, PA, NP, M, and NS) from H9N2 through reassortment, increasing their replication efficiency, host adaptation, and transmissibility in mammalian hosts [10–12].

Innate immunity is the first line of defence against viral infection. IAV entry activates host innate immunity by stimulating specific pattern recognition receptors (PRRs), such as Toll-like receptors (TLRs) [13], Nod-like receptors (NLRs) [14], and retinoic acid-inducible gene I-like receptors (RLRs) [15]. PRRs sense viral components and trigger a signalling cascade, which produces various cytokines, including type I and III interferons (IFNs) [16]. IFNs are critical components of innate immunity against IAV infection [16, 17]. IFNs bind to their receptors and activate JAK–STAT signalling, which induces the expression of antiviral proteins encoded by interferon-stimulated genes (ISGs) [18]. However, in some contexts, certain ISGs may cause harmful effects that contribute to inflammatory and autoimmune diseases [19, 20].

Long noncoding RNAs (lncRNAs) are transcripts exceeding 200 nucleotides in length that lack protein-coding potential [21]. They play crucial roles in the regulatory networks of various biological processes, including immunity, by modulating the expression of coding genes through mechanisms such as chromatin remodelling [22], transcriptional regulation [23], and posttranscriptional modifications [24, 25]. LncRNAs have been suggested to be involved in autoimmune disease, cancer progression, and viral pathogenesis [26–28]. Moreover, the expression of an increasing number of lncRNAs has been reported to be significantly influenced by viral infections and host–virus interactions [29, 30]. Some lncRNAs are exploited by viruses to promote disease progression, whereas others contribute to host defence against viral infection and are detrimental to viral survival [31]. LncRNAs are critical regulators of various aspects of antiviral innate immunity, including PRR-related signalling pathways, IFN and inflammatory cytokine production, JAK–STAT signalling, and the expression of antiviral ISGs [1]. Most lncRNAs have been studied in human and mammalian cells; for instance, the lncRNA NRAV acts as a negative regulator by altering histone modifications associated with genes of critical ISGs, thus suppressing their initial transcription [32]. Additionally, lncRNA-155, derived from MIR155HG, regulates host innate immunity during viral infection by modulating protein tyrosine phosphate 1B (PTP1B)-mediated interferon responses in human cells and mouse models [33]. However, the identification and function of lncRNAs in chickens remain largely unknown.

Recently, a study revealed a lncRNA array that plays a vital role in infectious bronchitis virus (IBV) pathogenicity in chickens [34]. Linc-satb1 was reported to play an important role in T-cell tumorigenesis induced by Marek’s disease virus (MDV) through the regulation of SATB1 protein-coding gene expression in chickens [35]. Linc-GALMD3 was found to positively *cis-*regulate downstream gaga-miR-233 gene expression, suggesting that it could be a key regulator in chickens and could be a promising marker for MD diagnosis, treatment, and prevention [36]. In addition, several lncRNAs, including MSTRG.14220.1, MSTRG.25416.43, MSTRG.24743.3, MSTRG.6458.31, and MSTRG.21445.2, are thought to play a role in IBV infection by regulating the innate immune response in chicken HD11 cells [34]. Despite their importance, the roles and underlying mechanisms of chicken lncRNAs in viral pathogenesis are poorly understood.

In this study, we investigated the involvement of chicken (*Gallus gallus*) lncRNAs in AIV infection and pathogenesis. RNA sequencing (RNA-seq) was conducted on chicken fibroblast (DF-1) cells either mock-infected or infected with the AIV H9N2 strain (A/Chicken/Fujian/MQ01/2015). The analysis revealed 2118 differentially expressed lncRNAs in H9N2-infected cells compared with control cells. Eight upregulated lncRNAs whose expression substantially increased were selected for further investigation. A lncRNA that we named lncRNA-up4 was particularly noteworthy, as it was significantly upregulated both in vitro and in vivo following infection with multiple influenza viral strains. Genomic analysis revealed that lncRNA-up4 is located upstream of the IL-6 gene. Importantly, functional analysis of lncRNA-up4 revealed that knockdown and overexpression of lncRNA-up4 significantly influenced viral replication. Furthermore, we found that lncRNA-up4 acts as an important negative regulator of innate immunity by suppressing the expression of type I IFN and several critical ISGs. It was also found to be involved in regulating the expression of several inflammatory cytokines at the mRNA level.

## Materials and methods

### Ethics statement

All animal experiments were approved by the Research Ethics Committee of Fujian Agriculture and Forestry University, China (permit no. PZCASFAFU2019009) and were performed under institutional guidelines for animal welfare.

### Cell lines and cell culture

Chicken DF-1, HD11 and human 293T cells were grown in Dulbecco’s modified Eagle’s medium (DMEM; Gibco, Grand Island, NY, USA) supplemented with 10% fetal bovine serum (FBS; Gibco, Grand Island, NY, USA), streptomycin, and penicillin (100 U/mL; Gibco, Grand Island, NY, USA) at 37 °C with 5% CO_2_ [32]. Moreover, cell passaging was conducted by digestion with 0.25% trypsin–EDTA every 24 h.

### Viruses and viral infection

Influenza A virus strains, H9N2, A/WSN/1933 (H1N1), and A/Puerto Rico/8/1934 (H1N1) were propagated in specific-pathogen-free embryonated chicken eggs following established protocols [1, 37]. DF-1 and HD11 cells were infected with the respective viruses (H9N2, WSN, and PR8) at a designated multiplicity of infection (MOI) of 1. After allowing viral adsorption for 1 h at 37 °C, the cells were rinsed with phosphate-buffered saline (PBS) and maintained in culture media for the indicated times (DMEM + 2 μg/mL trypsin).

### RNA sequencing

RNA-seq analysis was performed on DF-1 cells that were not infected or infected with H9N2 for 12 h (control and infected groups, respectively). A total of six samples were analysed, with three biological replicates in each group. Total RNA was extracted and subsequently evaluated for integrity with an Agilent 2100 bioanalyzer (Agilent Technologies, Santa Clara, CA, USA). The library was subsequently prepared with the TruSeq Stranded Total RNA kit with Ribo-Zero Gold, followed by sequencing on the Illumina platform. RNA sequencing and analysis were conducted by OE Biotech Co., Ltd. (Shanghai, China).

The raw reads obtained were subjected to a quality control process with FastQC, and adapter sequences and low-quality reads were trimmed with Trimmomatic. High-quality clean reads were aligned to the *Gallus gallus* reference genome (GRCg6a) with HISAT2. Transcript assembly and quantification were conducted with StringTie, and gene expression levels were normalized as fragments per kilobase of transcript per million mapped reads (FPKM).

Differential expression analysis between the infected and control groups was performed with DESeq2, with differentially expressed genes (DEGs) defined by an adjusted *p* value < 0.05 and a |log₂ fold change|≥ 1. Functional enrichment analysis of differentially expressed genes, including Gene Ontology (GO) terms and Kyoto Encyclopedia of Genes and Genomes (KEGG) pathway annotations, was conducted using the clusterProfiler package in R. The RNA-seq data have been deposited in the Gene Expression Omnibus (GEO) under accession number GSE279270.

### Generation of plasmids and stable cell lines

Short hairpin RNAs (shRNAs) specifically targeting TLR3, MDA5, NF-κB, or IRF7 were designed, cloned and inserted into a pSIH-H1-GFP vector. Lentiviruses expressing specific shRNAs targeting either lncRNA-up4 or luciferase control were used to transduce DF-1 and HD11 cells to generate stable knockdown cell lines. For overexpression studies, cells were transduced with pNL-EGFP carrying lncRNA-up4. Reporter plasmids, including IFNβ-Luc, NF-κB-Luc, pRL-TK, IL-6-Luc, and ISRE-Luc (purchased from Miaoling Biotechnology Co., Ltd., Wuhan, China), were used to analyse promoter activity. All transduced cell lines were subsequently infected with AIV or mock-infected for functional analyses.

### Cell stimulation

DF-1 cells were transfected with poly(I:C) (Invivogen, CA, USA) at different concentrations (0, 0.01, 0.1, and 1 µg/mL) with Lipofectamine 8000 (Beyotime, Biotech, China) for 4 h. To stimulate the cells with lipopolysaccharide (LPS from *E. coli* O111:B4), the cells were incubated with LPS (Peprotech, London, UK) at different concentrations (0, 2.5, 5, and 10 µg/mL) for 6 h. Bay 11–7082 (10 µM; Beyotime, Biotech, China), a specific inhibitor of NF-κB, was used to analyse the function of NF-κB in the regulation of lncRNA-up4 expression. Dimethyl sulfoxide (DMSO) (Sigma‒Aldrich, Germany) was used as a solvent for Bay 11–7082 and served as a vehicle control in parallel treatments. All treatments were performed according to the manufacturer’s guidelines.

### Subcellular fractionation

Subcellular fractionation experiments were performed with the PARIS™ Kit Protein and RNA Isolation System (Invitrogen, Thermo Fisher Scientific) according to the manufacturer’s instructions.

### Plaque-forming and haemagglutinin assays

MDCK cells were incubated with serially diluted supernatants collected from virus-infected, transduced DF-1 cell cultures at 37 °C for 1 h. After infection, the cells were washed with PBS and overlaid with 3% low-melting agarose (Promega, WI, USA), which was prepared by mixing agarose with phenol red-free DMEM (high glucose, HEPES, without phenol red) (Gibco, USA) at a ratio of approximately 1:4. The overlay was supplemented with penicillin (100 U/mL; Gibco, Grand Island, NY, USA), streptomycin (100 U/mL; Gibco, Grand Island, NY, USA), tetracycline (10 μg/mL; Sigma‒Aldrich, St. Louis, MO, USA) and L-1-tosylamido-2-phenylethyl chloromethyl ketone (TPCK)-treated trypsin (2 µg/mL; Sigma‒Aldrich, St. Louis, MO, USA). The cells were incubated upside-down at 37 °C for 72 h, followed by staining with 1% crystal violet hydrate solution, after which the plaques were manually counted. For the haemagglutination assay (HA), the supernatants were serially diluted in PBS and mixed with a 1% chicken erythrocyte suspension. Viral titers were subsequently determined from the highest dilution that resulted in a positive haemagglutination reaction.

### RT‒PCR and quantitative real-time PCR (qRT‒PCR)

Total RNA was extracted from DF-1 and HD11 cells with TRIzol reagent (TIANGEN, Beijing, China). cDNA was synthesized by a HiScript III 1st Strand cDNA Synthesis Kit (Vazyme, Nanjing, China). RT‒PCR was conducted with Taq DNA polymerase (GenStar, Beijing, China), while qRT‒PCR was performed with SYBR Green Master Mix (Vazyme, Nanjing, China). The qRT‒PCR data are presented as autocalculated normalized ratios determined through the 2^−ΔΔCT^ method with the Light Cycler 96 system (Roche, Basel, Switzerland). The primer sequences and corresponding gene accession numbers are listed in Additional file 1.

### Western blotting

After the experimental treatments were performed, the cells were harvested, and lysates were prepared. SDS‒PAGE and western blotting were conducted as previously described [33]. Briefly, proteins were separated by SDS‒PAGE, transferred onto a nitrocellulose membrane that was then blocked in TBS buffer (10 mM Tris–HCl, pH 7.4; 150 mM NaCl) containing 5% skim milk and subsequently incubated with primary antibodies. The following antibodies were used: anti-IAV NP (generated in our laboratory; dilution 1:20 000) and anti-β-actin (1:8000; TransGen Biotech, Beijing, China). The protein bands were detected by chemiluminescence with a FluorChem E System (ProteinSimple, San Jose, CA, USA).

### Animal experiments

Seven-day-old SPF White Leghorn chickens were divided into control and H9N2-infected groups (*n* = 6). Chickens were intranasally inoculated with 10^6^ TCID_50_ of H9N2 virus in 200 µL of PBS. At 48 h post-infection, all the chickens were euthanized by intravenous sodium pentobarbital (100 mg/kg) following institutional animal care guidelines. Lung, trachea, spleen, liver, heart, kidney, and intestine were aseptically collected for the examination of lncRNA expression and viral replication. Total RNA was extracted with TRIzol reagent (TIANGEN, China) and analysed by RT‒PCR. Protein samples were analysed by western blotting.

### Statistical analysis

Statistical analysis was performed with GraphPad Prism software (GraphPad Software, San Diego, CA, USA). Significant differences between groups were determined by Student’s *t* test to compare two groups. The data are reported as the mean ± standard deviation (SD), and *p* < 0.05 was considered to indicate statistical significance.

## Results

### IAV infection significantly induces lncRNA-up4 expression in vitro and in vivo

**Figure 1:**
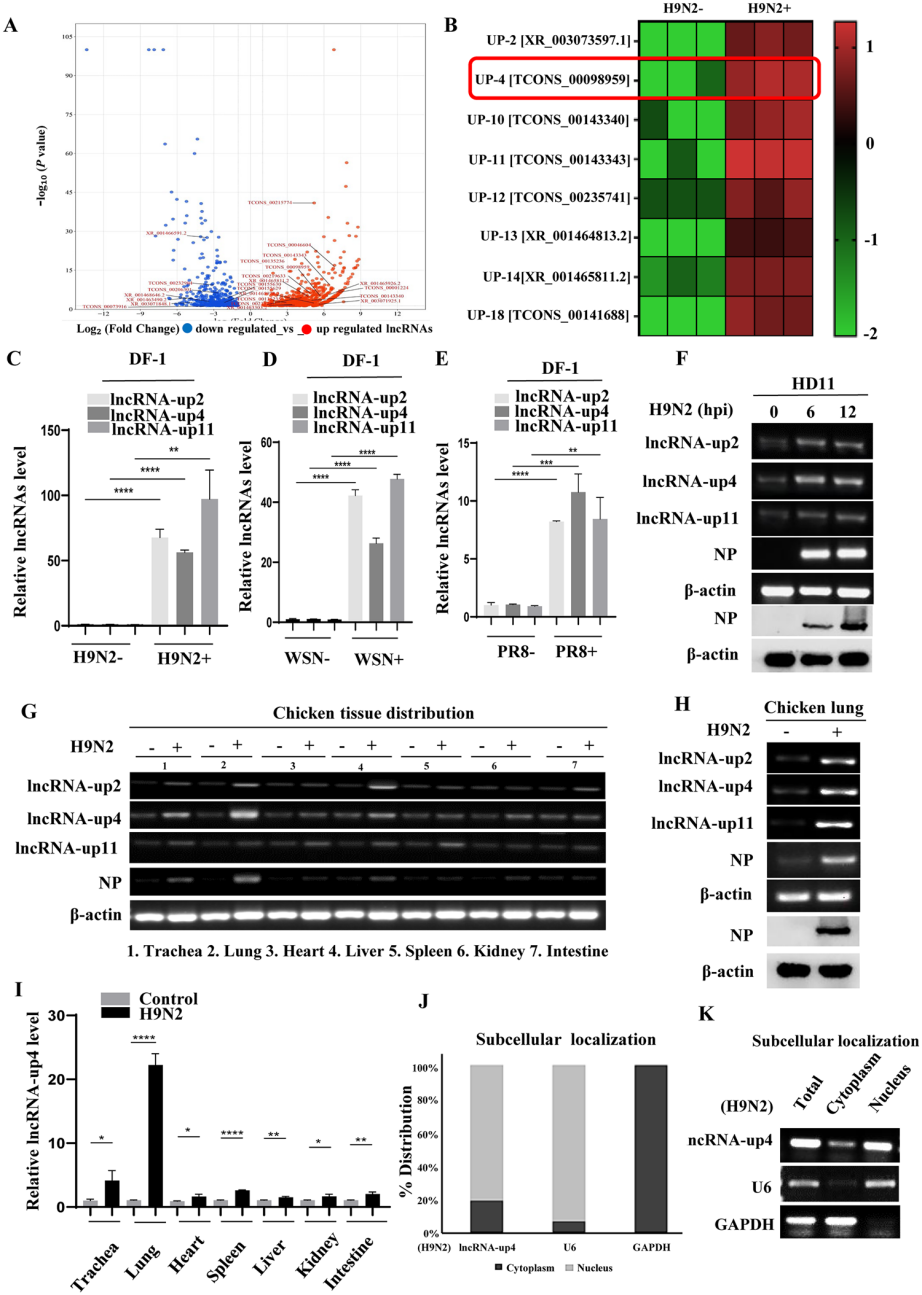
**IAV infection significantly increased lncRNA-up4 expression both in vitro and in vivo.** **A** Volcano plot analysis of RNA-seq data (GSE279270) from H9N2-infected DF-1 cells revealed 2118 differentially expressed lncRNAs compared with the control (1559 upregulated and 559 downregulated; *P* value < 0.05, and |log_2_FC|> 1). **B** Heatmap of eight highly upregulated lncRNAs (*P* < 0.05, |log_2_FC|> 2) with potential immune relevance. **C–E** qRT‒PCR validation of lncRNA-up2, lncRNA-up4, and lncRNA-up11 expression in DF-1 cells after infection with the H9N2, WSN, and PR8 IAV strains. **F** Time course expression of lncRNAs in HD11 cells at 0, 6, and 12 h post-infection, showing progressive upregulation. **G** In vivo RT‒PCR analysis confirming lncRNA induction in multiple chicken tissues after H9N2 infection. **H** Increased expression of lncRNA-up2, lncRNA-up4, and lncRNA-up11 in H9N2-infected chicken lung tissue. **I** Tissue distribution analysis by qRT‒PCR revealed the highest lncRNA-up4 expression in the lung post-infection. **J**, **K** Subcellular localization showing predominant nuclear enrichment (~80%) of lncRNA-up4, with ~20% in the cytoplasm. All RT‒PCR and qRT‒PCR results are representative of three independent experiments with similar results. The data are presented as the mean ± SD. ^*^p < 0.05, ^**^p < 0.01, ^***^p < 0.001, ^****^p < 0.0001.

To investigate the functional involvement of chicken lncRNAs in AIV infection and pathogenesis, we conducted RNA-seq analysis of H9N2-infected DF-1 cells (GEO accession number: GSE279270). The analysis revealed 2118 differentially expressed lncRNAs (1559 upregulated, 559 downregulated; H9N2 infection vs. control,* P* value < 0.05 and |log_2_FC|> 1). Volcano plot analysis confirmed the presence of many upregulated lncRNAs in infected cells, suggesting their involvement in IAV infection (Figure 1A). Principal component analysis (PCA) revealed distinct clustering of the infected and control groups, demonstrating consistent and reproducible virus-induced gene expression patterns across biological replicates (Additional file 2A). Gene Ontology (GO) analysis of the differentially expressed lncRNAs revealed strong enrichment in immune-related biological processes (Additional file 2B). To identify key lncRNAs involved in IAV infection and pathogenesis, we screened highly upregulated lncRNAs that potentially modulate IAV infection. Eight highly upregulated lncRNAs were subsequently selected on the basis of their fold change (*P* value < 0.05 and |log_2_FC|> 2) and are displayed as a heatmap (Figure 1B). Among them, three lncRNAs, lncRNA-up2 (XR_003073597.1), lncRNA-up4 (TCONS_00098959) and lncRNA-up11 (TCONS_00143343), were significantly upregulated (fold change > 2) upon infection with the H9N2, PR8, and WSN IAV strains in DF-1 cells (Figures 1C–E; Additional file 2C).

Furthermore, time-course experiments revealed the progressive upregulation of the expression of these lncRNAs in HD11 cells at different times postinfection, suggesting their potential role in response to IAV challenge (Figure 1F; Additional files 2D–G). To validate the in vivo relevance, 7-day-old chickens were infected with H9N2 AIV, and the expression of lncRNA-up2, lncRNA-up4, and lncRNA-up11 was examined by RT‒PCR. Consistent with the in vitro results, the in vivo results revealed that expression of these lncRNAs clearly increased in the lungs and trachea after viral infection (Figures 1G and H). Consistently, viral NP expression was predominantly detected in the lungs and trachea, indicating active viral replication in these tissues (Figure 1G). LncRNA expression was also slightly upregulated in other organs, including the liver and spleen, of the animals infected with H9N2 AIV (Figure 1G). LncRNA-up4 was selected for further study because of its strong upregulation in vitro and in vivo and its genomic analysis, which revealed that lncRNA-up4 was a 2214 bp transcript from coding DNA located upstream of the IL-6 gene on chicken chromosome number 2.

Next, the expression of lncRNA-up4 and viral NP was examined in several chicken tissues infected with AIV or mock-infected. We detected lncRNA-up4 in the trachea, lung, heart, liver, kidney, spleen, and intestine (Figure 1I). In particular, the expression of lncRNA-up4 was the most significantly increased in the lung after H9N2 infection, suggesting that lncRNA-up4 was functionally involved in the response to AIV infection (Figure 1I). Next, a subcellular fractionation experiment revealed that lncRNA-up4 was present in both the nucleus and the cytoplasm but was located primarily in the nucleus, as demonstrated by qRT‒PCR and RT‒PCR, with U6 snRNA as a nuclear control (Figures 1J, K; Additional file 2H). H9N2 infection slightly increased its nuclear enrichment (Additional file 2H). Together, these results suggest that lncRNA-up4 is an IAV-induced lncRNA that may be involved in viral replication and pathogenesis.

### Virus-induced lncRNA-up4 expression is regulated by PRR-dependent innate immune signalling

**Figure 2:**
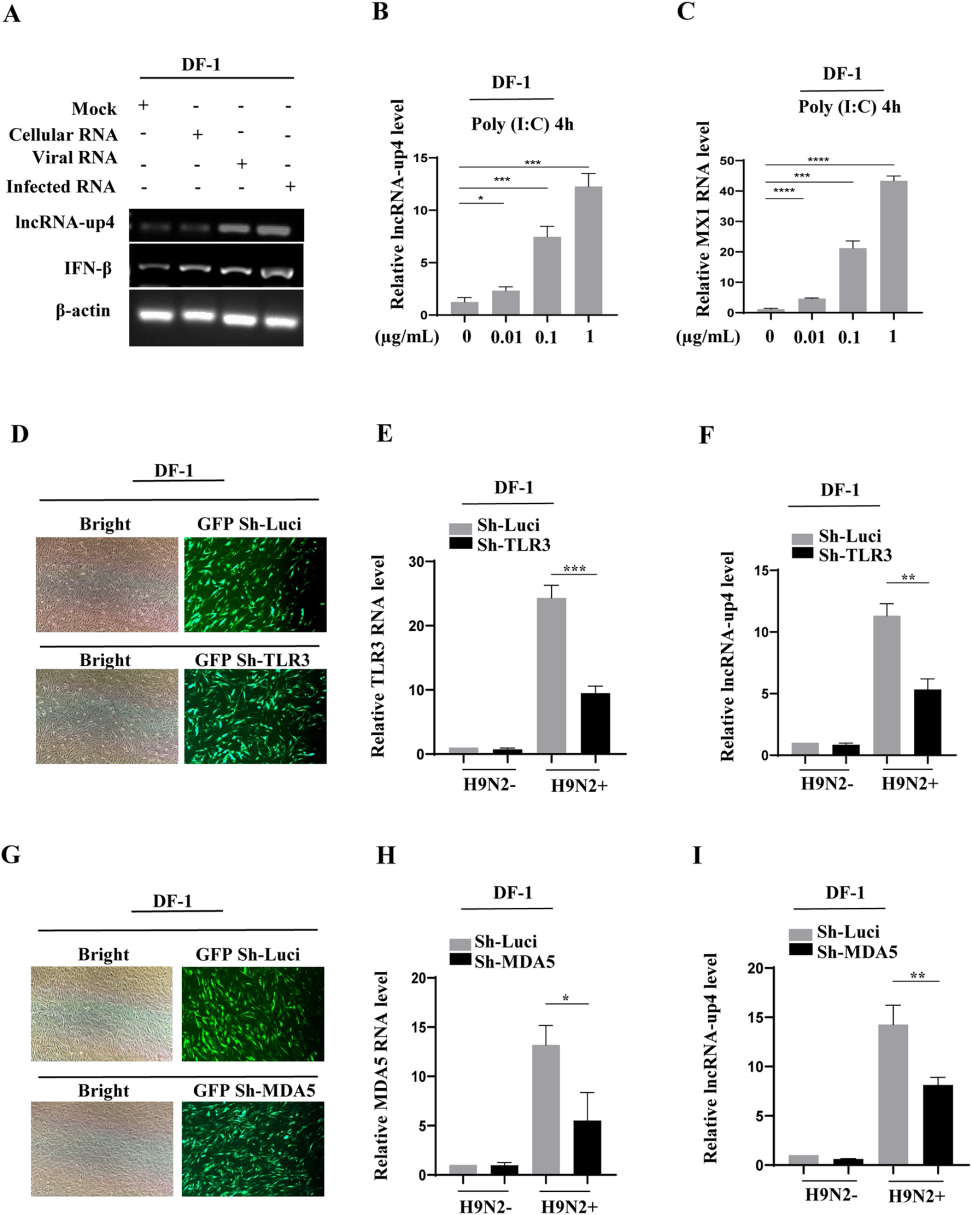
**Virus-induced lncRNA-up4 expression is regulated by PRR-dependent innate immune signalling**.** A** RT‒PCR analysis showing significant induction of lncRNA-up4 expression in DF-1 cells treated with viral RNA and RNA from infected cells compared with cells treated with control cellular RNA. **B**, **C** Poly(I:C) stimulation led to dose-dependent upregulation of lncRNA-up4 expression, as validated by qRT‒PCR. MX1 expression confirmed the poly(I:C) activation of antiviral signalling. **D–F** Transduction efficiency was confirmed by GFP fluorescence in cells transduced with shRNAs targeting TLR3 (**D**). TLR3 knockdown significantly reduced lncRNA-up4 expression in DF-1 cells after H9N2 infection, as shown by qRT‒PCR (E, F). **G–I** The transduction efficiency was confirmed by GFP fluorescence in cells transduced with shRNAs targeting MDA5 (**G**). MDA5 knockdown partially impaired virus-induced lncRNA-up4 expression, as validated by qRT‒PCR (**H**, **I**). All RT‒PCR and qRT‒PCR results are representative of three independent experiments with similar results. The data are presented as the mean ± SD. ^*^p < 0.05, ^**^p < 0.01, ^***^p < 0.001, ^****^p < 0.0001.

We next sought to investigate the signalling pathway that governs lncRNA-up4 induction by viral infection. First, DF-1 cells were treated with viral genomic RNA (viral RNA), RNA derived from infected cells (infected RNA) or cellular RNA from noninfected cells as a control to investigate how lncRNA-up4 was specifically induced by viral infection. The results revealed that compared with the mock treatment, viral RNA and infected RNA induced lncRNA-up4 expression (Figure 2A). As expected, IFN-β, which was used as a positive control for the antiviral response, was also induced under the same conditions, confirming effective stimulation of the innate immune pathway. These results indicate that lncRNA-up4 expression can be triggered by viral RNA or replication-associated intermediates during H9N2 infection.

During IAV infection, conserved pathogen-associated molecular patterns (PAMPs) are recognized by PRRs, which activate a series of signalling cascades that induce various molecules involved in innate immunity [38]. Poly(I:C) is a synthetic analogue of viral double-stranded RNA that can be detected by PRRs and trigger a signalling cascade. Thus, we treated DF-1 cells with different concentrations of poly(I:C) and evaluated lncRNA-up4 expression. The results revealed a gradual increase in the expression of lncRNA-up4 in a poly(I:C) dose-dependent manner, as evidenced by the RT‒PCR and qRT‒PCR data (Figure 2B; Additional file 3A). MX1 gene expression was used to validate the function of poly(I:C) (Figure 2C; Additional file 3A).

Finally, we investigated the involvement of key PRRs, including TLR3 and MDA5, in the regulation of lncRNA-up4 expression in DF-1 cells during H9N2 infection. To assess the role of TLR3 and MDA5 in lncRNA-up4 induction, we performed targeted knockdown experiments in DF-1 cells with shRNA-mediated silencing. Specific shRNA constructs against TLR3 or MDA5 were delivered via lentiviral transduction, and the transduction efficiency was examined via GFP fluorescence visualization (Figures 2D, G). We observed that lncRNA-up4 expression was significantly reduced by TLR3 knockdown in DF-1 cells infected with the H9N2 virus (Figures 2E, F; Additional file 3B). MDA5 knockdown also significantly impaired the virus-induced expression of lncRNA-up4 (Figures 2H, I; Additional file 3C). Together, these results suggest that lncRNA-up4 expression is regulated by a PRR-dependent signalling pathway during viral infection.

### NF-κB-mediated signalling plays a key role in the regulation of virus-induced lncRNA-up4 expression

**Figure 3:**
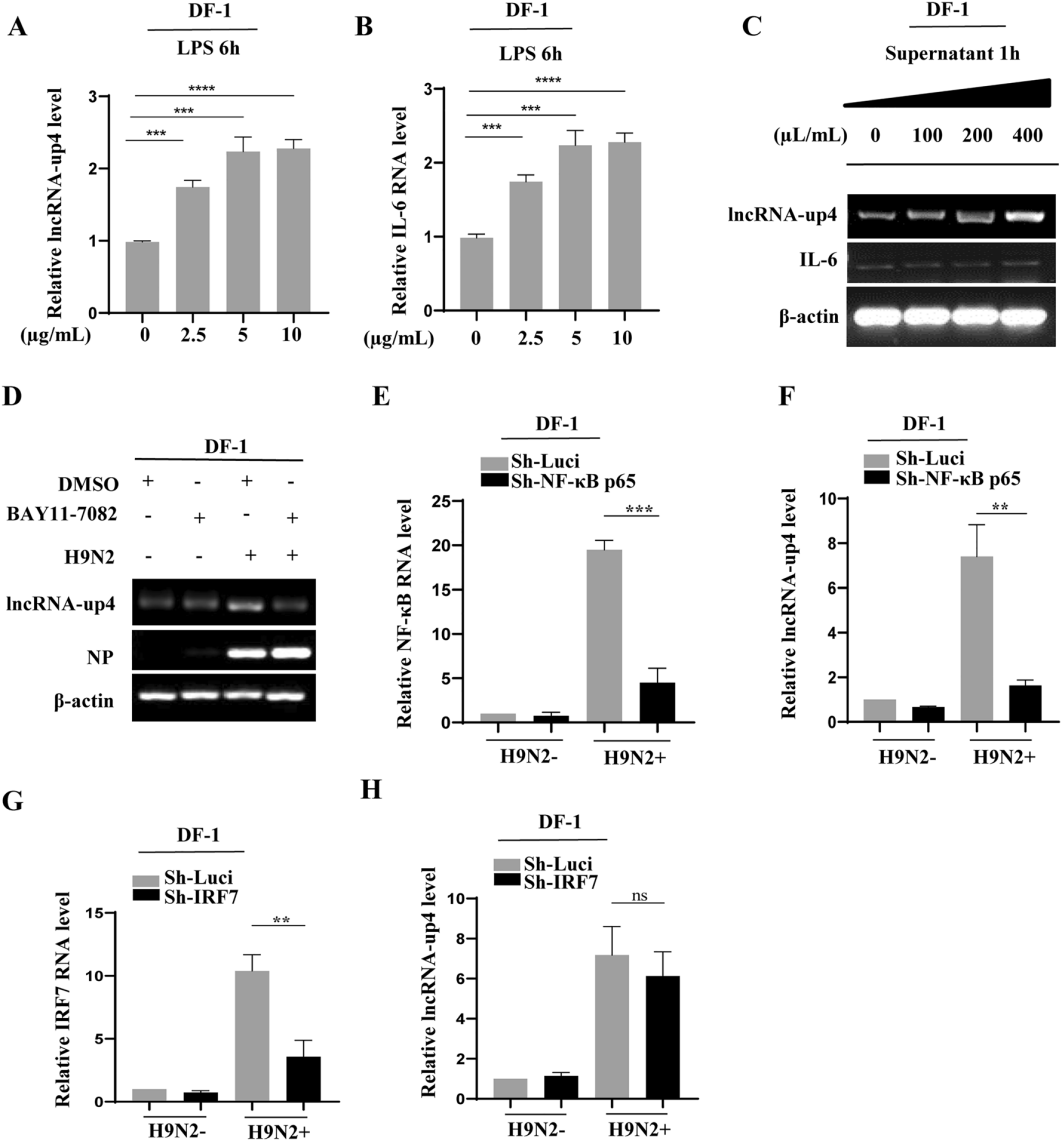
**NF-κB-mediated signalling plays a key role in the regulation of virus-induced lncRNA-up4 expression**.** A**, **B** At 6 h, LPS stimulation led to dose-dependent increases in lncRNA-up4 and IL-6 mRNA expression in DF-1 cells, as validated by qRT‒PCR. **C** DF-1 cells were treated with LPS-stimulated supernatants. This treatment increased lncRNA-up4 expression, while IL-6 expression remained unchanged. **D** RT‒PCR analysis of lncRNA-up4 expression in DF-1 cells treated with Bay 11–7082 or the DMSO control and infected with H9N2. **E**, **F** DF-1 cells were transduced with sh-NF-κB p65 or negative control sh-Luci and infected with H9N2. At 12 h post-infection, the expression of lncRNA-up4 and NF-κB p65 was determined by qRT‒PCR. **G**, **H** qRT‒PCR analysis of lncRNA-up4 and IRF7 expression in DF-1 cells following IRF7 knockdown and infection with H9N2. All RT‒PCR and qRT‒PCR results are representative of three independent experiments with similar results. The data are shown as the mean ± SD. ^**^p < 0.01, ^***^p < 0.001, ^****^p < 0.0001, and ns represents no significance.

Because the results revealed that PRR-dependent signalling pathway(s) play a critical role in regulating virus-induced lncRNA-up4 expression, we further examined the downstream signalling pathway molecules to determine their effects on lncRNA-up4 expression. First, LPS, a strong activator of NF-κB-mediated signalling, was used. DF-1 cells were treated with increasing concentrations of LPS. Interestingly, we observed significant upregulation of lncRNA-up4 expression in a dose-dependent manner (Figure 3A; Additional file 4A). As a positive control, IL-6 mRNA expression was induced by LPS treatment, as expected (Figure 3B; Additional file 4A). The genomic sequence of lncRNA-up4 is located upstream of the interleukin 6 (IL-6) gene, a key cytokine involved in the inflammatory response associated with IAV infection. To address the relationship between lncRNA-up4 and the IL-6 gene, we treated DF-1 cells with cytokine-containing supernatants derived from LPS-stimulated DF-1 cells at different concentrations for 1 h. The results revealed an increase in lncRNA-up4 expression, whereas IL-6 expression remained unchanged by treatment with the supernatants (Figure 3C). Second, DF-1 cells were treated with Bay 11–7082, a well-known inhibitor of the NF-κB signalling cascade. The data revealed dramatically decreased lncRNA-up4 expression after Bay 11–7082 treatment following virus infection compared with that in the control (Figure 3D). Similar results were obtained from HD11 cells with the same treatment (Additional file 4B). Next, we used shRNA-mediated NF-κB p65 knockdown to confirm these findings. As shown in Figures 3E, F and Additional files 4C–D, disruption of NF-κB p65 expression caused a significant decrease in lncRNA-up4 expression in cells infected with H9N2 virus, as evidenced by RT‒PCR and qRT‒PCR data.

Furthermore, we investigated the involvement of IRF7, another critical transcription factor for innate immunity, including the type I IFN response to IAV infection, in the regulation of lncRNA-up4 expression during H9N2 infection. DF-1 cells were transduced with shRNA targeting IRF7, and the results revealed that lncRNA-up4 expression was only slightly reduced in IRF7 knockdown cells challenged with H9N2 (Figures 3G, H; Additional files 4E–F). Together, these results suggest that virus-induced lncRNA-up4 expression is likely regulated by the NF-κB-mediated pathway downstream of PRR-dependent innate immune signalling.

### Silencing lncRNA-up4 significantly impedes IAV replication

**Figure 4:**
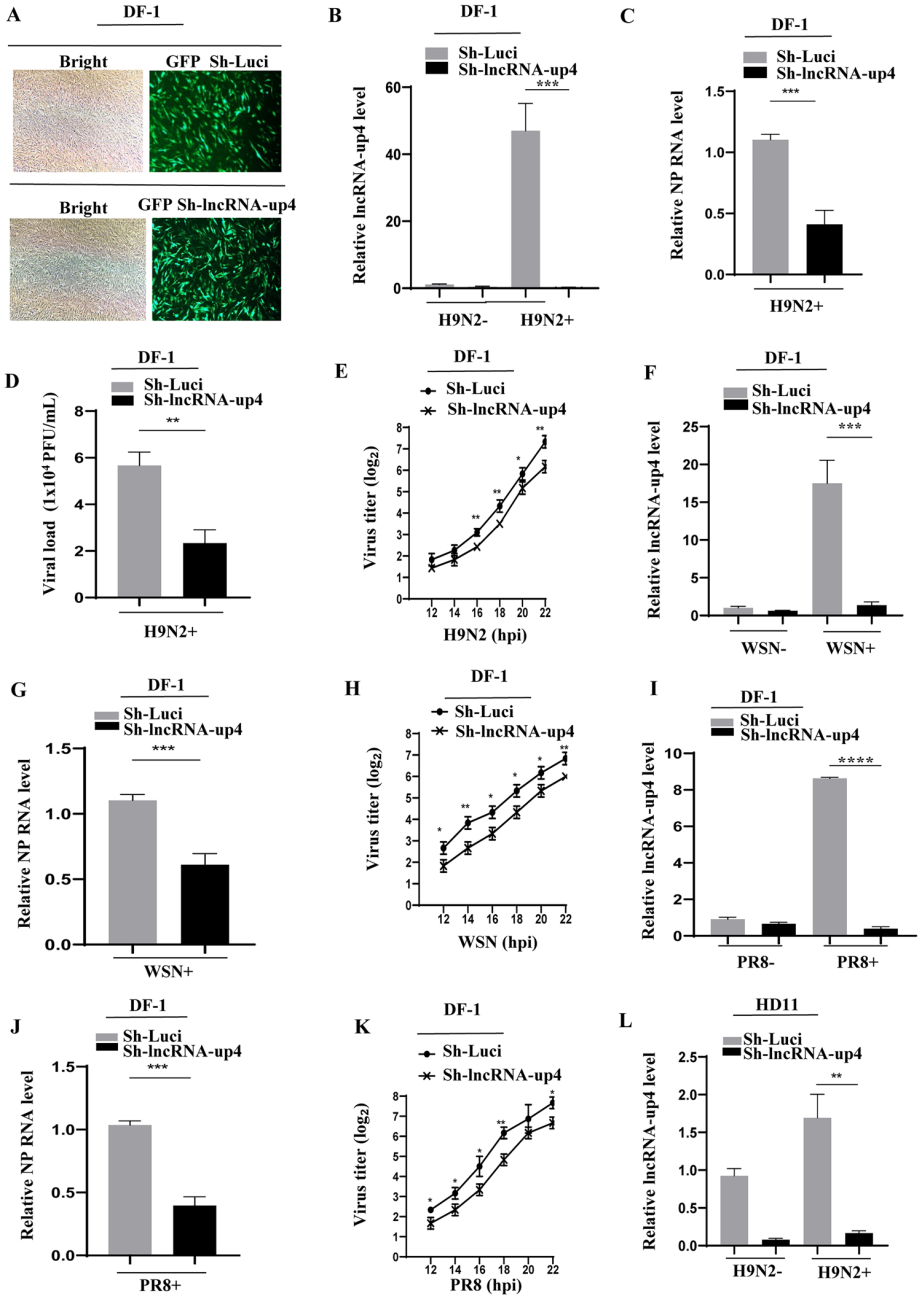
**Silencing lncRNA-up4 expression significantly impedes IAV replication**.** A** Fluorescence microscopy confirmed the successful transduction of DF-1 cells with shRNA targeting lncRNA-up4 or a luciferase control. **B**, **C** qRT‒PCR analysis revealed a significant reduction in lncRNA-up4 expression and H9N2 viral replication in lncRNA-up4 knockdown cells. **D**, **E** Viral load and titer were significantly reduced in lncRNA-up4 knockdown cells, as assessed by PFA and HA assays. **F–K** Consistent reductions in lncRNA-up4 and NP mRNA expression, along with impaired viral replication kinetics (HA titration), were observed in lncRNA-up4 knockdown cells infected with WSN and PR8 IAV strains. **L** The viral titer was significantly reduced in lncRNA-up4-knockdown HD11 cells, as assessed by the HA assay. The data are presented as the mean ± SD. All RT‒PCR and qRT‒PCR results are representative of three independent experiments with similar results. ^**^p < 0.01, ^***^p < 0.001.

To determine the role of lncRNA-up4 in IAV replication, DF-1 cells were stably transduced with shRNA targeting lncRNA-up4 or luciferase control. By evaluating GFP expression through fluorescence microscopy, successful transduction was confirmed (Figure 4A). Next, we validated the lncRNA-up4 knockdown efficiency in cells infected with H9N2 AIV or mock-infected, and the results revealed a marked reduction in the lncRNA-up4 level in the specific shRNA-expressing cells compared with that in the luciferase control cells, as measured by RT‒PCR and qRT‒PCR (Figure 4B; Additional file 5A). Furthermore, the effect of silencing lncRNA-up4 on H9N2 replication was investigated. We observed that viral nucleoprotein (NP) mRNA expression was significantly lower in lncRNA-up4 knockdown cells than in luciferase control cells (Figure 4C; Additional file 5A). Mock-infected controls were also included to confirm infection specificity. Moreover, the viral load was determined by a plaque-forming assay (PFA), and the results revealed a significant reduction in viral load in the knockdown cells compared with that in the control cells (Figure 4D). Viral replication kinetics were also evaluated by an HA assay. Supernatants from the transduced cell lines were collected at various time points post-infection and subjected to titration. Consistent with the PFA results, the HA assay results revealed a significant reduction in viral replication in lncRNA-up4-knockdown cells compared with that in control cells (Figure 4E).

In addition, we used two other strains, namely, IAV WSN and PR8, to determine the effect of lncRNA-up4 on viral replication. Similar trends in viral NP mRNA and protein expression were observed in lncRNA-up4 knockdown cells following infection with WSN or PR8 IAV strains, as determined by RT‒PCR, western blotting, and qRT‒PCR (Figures 4F‒G and 4I‒J; Additional files 5B and C). Additionally, the viral replication kinetics in lncRNA-up4 knockdown and control cells after WSN and PR8 infection were evaluated by HA assays at the indicated time points post-infection. As expected, an obvious decrease in viral replication was detected in the knockdown cells compared with the control cells (Figures 4H, K).

To confirm the above findings, the HD11 cell line was used. Indeed, a significant reduction in lncRNA-up4, NP mRNA and viral protein expression was detected in the knockdown cells compared with the control cells (Additional files 5D–F). Additionally, the results of the HA assay at the indicated time points post-infection confirmed that H9N2 replication was suppressed by silencing lncRNA-up4 in HD11 cells (Figure 4L). Taken together, our data support an important role for lncRNA-up4 in the upregulation of viral replication.

### Overexpression of lncRNA-up4 increases IAV replication

**Figure 5:**
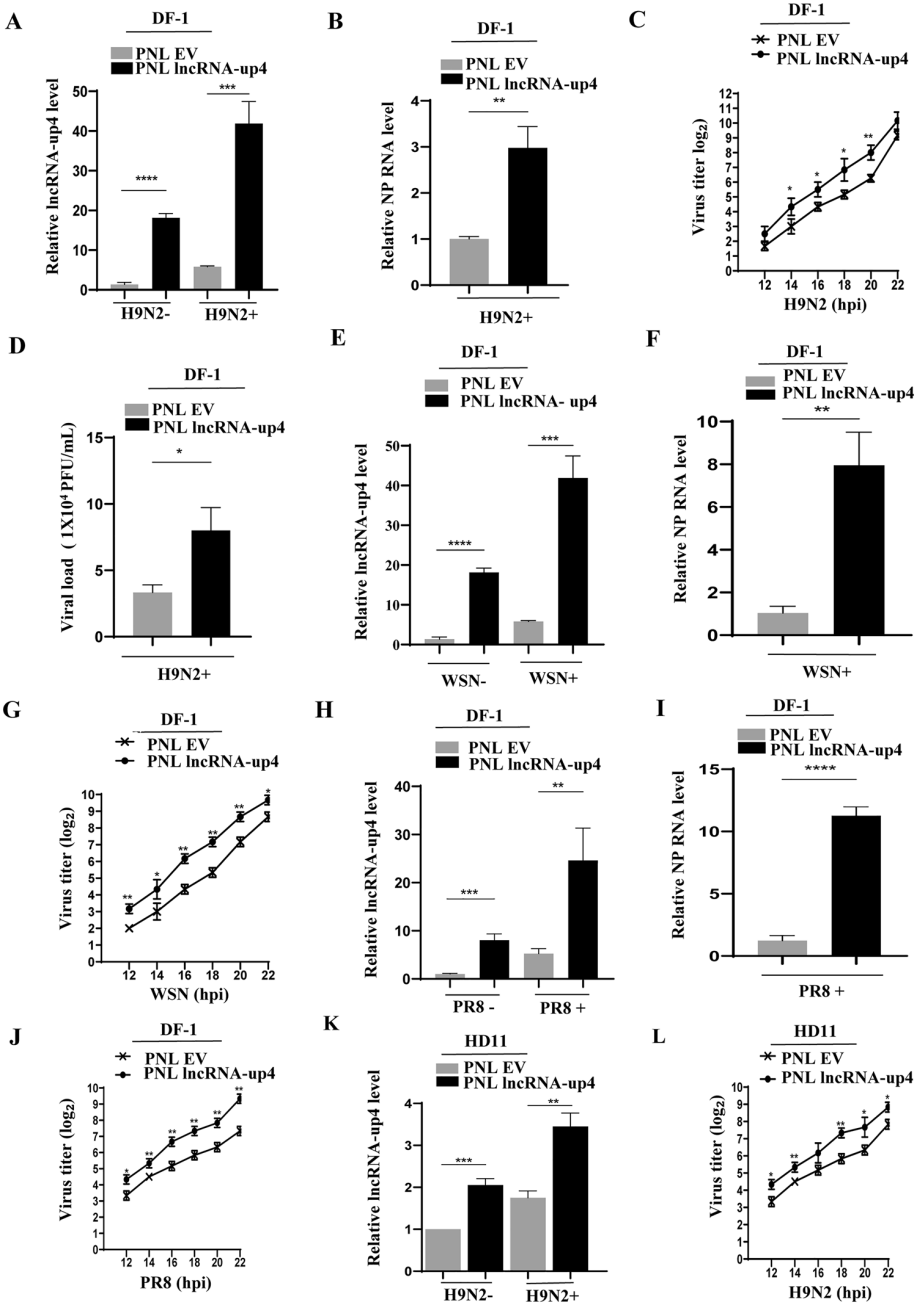
**Overexpression of lncRNA-up4 increases IAV replication**.** A**, **B** Overexpression of lncRNA-up4 in DF-1 cells transduced with the lncRNA-up4 expression plasmid or empty vector was confirmed by qRT‒PCR. Viral NP mRNA and lncRNA-up4 expression in these cells following H9N2 infection was demonstrated by qRT‒PCR. **C**, **D** HA and PFA assays revealed increased viral replication in overexpressing cells compared with that in control cells. **E–J** Increased lncRNA-up4 mRNA and NP mRNA in overexpressing cells following infection with WSN and PR8 viral strains, which was further confirmed by higher viral titers in HA assays. **K**, **L** qRT‒PCR was used to validate lncRNA-up4 overexpression in HD11 cells, and HA assays confirmed increased viral titers upon H9N2 infection. All the results represent three independent experiments with similar findings. The data are presented as the mean ± SD. ^*^p < 0.05, ^**^p < 0.01, ^***^p < 0.001.

On the other hand, we constructed a lncRNA-up4 overexpression plasmid by using the pNL-EGFP vector to further confirm the functional involvement of this lncRNA in the regulation of IAV replication. Stable DF-1 cell lines overexpressing lncRNA-up4 or the PNL empty vector (PNL-EV) were generated. The overexpression efficiency of lncRNA-up4 was verified by RT‒PCR and qRT‒PCR (Figure 5A; Additional file 6A). These cells were then mocked-infected or infected with H9N2, and RNA samples were collected to examine lncRNA-up4 and viral NP expression. Both RT‒PCR and qRT‒PCR results validated the higher levels of expression of viral NP mRNA in lncRNA-up4-overexpressing cells than in control cells (Figure 5B; Additional file 6A). Simultaneously, total proteins were harvested, and western blotting was performed to assess the viral NP protein. NP protein expression was substantially greater in the overexpression cells than in the control cells (Additional file 6A).

Furthermore, HA and PFA assays were conducted to provide more evidence of the effect of lncRNA-up4 overexpression on viral replication. Compared with the control, lncRNA-up4 overexpression increased viral production in the cells (Figures 5C, D). Moreover, we evaluated viral NP expression by RT‒PCR, qRT‒PCR, and western blotting to determine whether lncRNA-up4 had any effect on WSN infection. We observed that lncRNA-up4 overexpression significantly increased viral NP expression at both the mRNA and protein levels (Figures 5E, F; Additional file 6B). As quantified by the HA assay, supernatants from the challenged cells demonstrated higher viral titers in the lncRNA-up4 overexpressing cells compared with the control cells (Figure 5G). Additionally, the stable overexpression cell lines were mock-infected or infected with the PR8 strains of IAV. Similarly, we noticed a clear increase in viral NP expression at both the mRNA and protein levels in the overexpression cells compared with the control cells (Figures 5H, I; Additional file 6C). HA assays were conducted following PR8 IAV infection, and the results confirmed the upregulation of viral replication in lncRNA-up4-overexpressing cells compared with that in control cells (Figure 5J).

We also utilized HD11 cells to verify the above observations. The cells were transduced with the lncRNA-up4 overexpression vector, followed by H9N2 infection. The efficiency of lncRNA-up4 overexpression in HD11 cells was determined by RT‒PCR and qRT‒PCR (Figure 5K; Additional file 6D). Similarly, lncRNA-up4 overexpression resulted in marked increases in the levels of the NP mRNA and protein (Additional files 6D–E). HA assay analysis revealed higher viral titers in the lncRNA-up4-overexpressing cells, supporting its role in increasing IAV replication (Figure 5L). These observations reveal a potential association between lncRNA-up4 and IAV replication.

### LncRNA-up4 knockdown has different effects on the expression of various cytokines and interferon-stimulated antiviral genes

**Figure 6:**
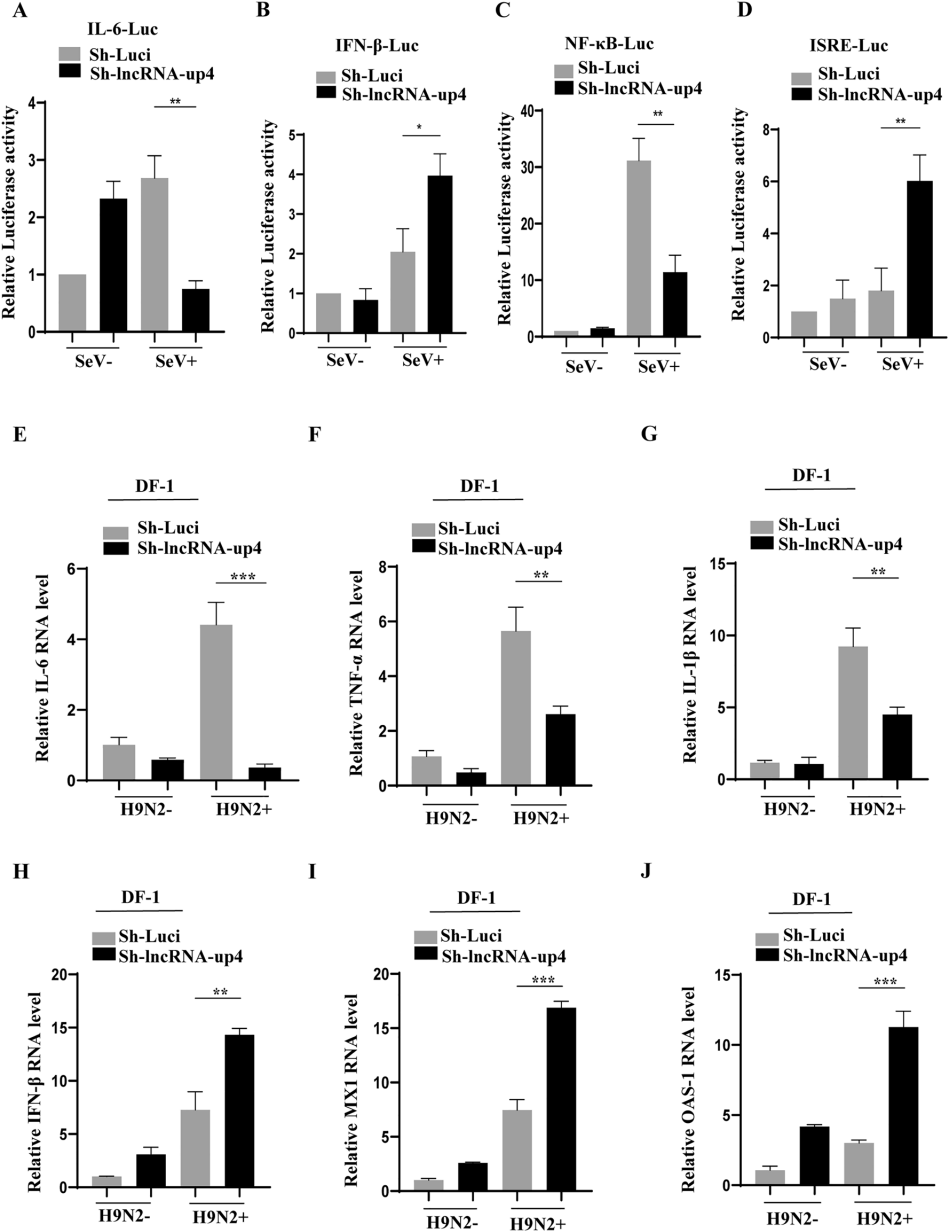
**LncRNA-up4 knockdown has different effects on the expression of various cytokines and interferon-stimulated antiviral genes**.** A–D** Luciferase reporter assays in lncRNA-up4 knockdown and control DF-1 cells revealed reduced IL-6 and NF-κB promoter activity (**A**, **C**) but increased IFN-β and ISRE-mediated transcription (B, D) following viral infection. **E–G** qRT‒PCR demonstrated a significant reduction in IL-6, TNF-α and IL-1β mRNA expression in lncRNA-up4 knockdown cells with H9N2 infection. **H–J** IFN-β, MX1, and OAS-1 mRNA levels were significantly upregulated in lncRNA-up4-knockdown cells compared with those in control cells. All the results represent three independent experiments with similar findings. The data are presented as the mean ± SD. ^*^p < 0.05, ^**^p < 0.01, ^***^p < 0.001.

To further address the relationship between lncRNA-up4 and innate antiviral responses, we used a luciferase reporter system with a lncRNA-up4 knockdown stable cell line and control cells infected with Sendai virus (SeV) or mock-infected as previously described [39]. With this system, we examined the effects of lncRNA-up4 knockdown on the transcription or promoter activation of IL-6, IFN-β, NF-κB, and ISRE. Luciferase analysis of the promoter interaction revealed significantly lower IL-6 luciferase activity after viral infection in the knockdown cells than in the control cells (Figure 6A). Next, the IFN-β-luciferase reporter system was tested in lncRNA-up4 knockdown cells and control cells. Interestingly, the results revealed that IFN-β luciferase activity was significantly greater in the lncRNA-up4 knockdown cell line than in the control cell line (Figure 6B), suggesting that lncRNA-up4 may act as a negative regulator of type I interferon signalling. An NF-κB-luciferase reporter system was then used, and the lncRNA-up4 knockdown cell line and control cell line were transfected with the reporter DNA. We detected a significant downregulation of the NF-κB-mediated response in the knockdown cell line (Figure 6C). In addition, to determine the effect of lncRNA-up4 on the transcriptional activation of ISRE, the ISRE-luciferase system was selected for further investigation. A substantial increase in ISRE-mediated luciferase activity was observed in the knockdown cells (Figure 6D). These results indicate that lncRNA-up4 may promote an inflammatory response but suppress the IFN-mediated immune response during viral infection.

The coding sequence of lncRNA-up4 is located near the gene encoding IL-6, an important inflammatory cytokine. Thus, we investigated whether the expression of IL-6 could be influenced by lncRNA-up4. To test this possibility, lncRNA-up4 knockdown and control DF-1 cells were infected with H9N2 virus or mock-infected, and the expression of IL-6 was measured. Interestingly, a significant reduction in IL-6 mRNA expression was observed in the lncRNA-up4 knockdown cell line compared with that in the control (Figure 6E). Furthermore, we examined several other cytokines, including TNF-α, IL-1β and IFN-β, as well as critical ISGs, such as MX1 and OAS-1, that play crucial roles in innate immunity to investigate the effect of lncRNA-up4 knockdown on their mRNA expression. We observed that knocking down lncRNA-up4 expression significantly decreased TNF-α and IL-1β mRNA expression in cells upon H9N2 AIV infection (Figures 6F, G). In contrast, the expression of IFN-β, MX1, and OAS-1 at the mRNA level was significantly greater in lncRNA-up4 knockdown cells than in control cells infected with H9N2 IAV (Figures 6H–J). These results suggest that lncRNA-up4 may regulate viral replication by altering the expression of multiple cytokines and some critical ISGs that contribute to innate antiviral responses.

### Overexpression of lncRNA-up4 impairs the antiviral response

**Figure 7:**
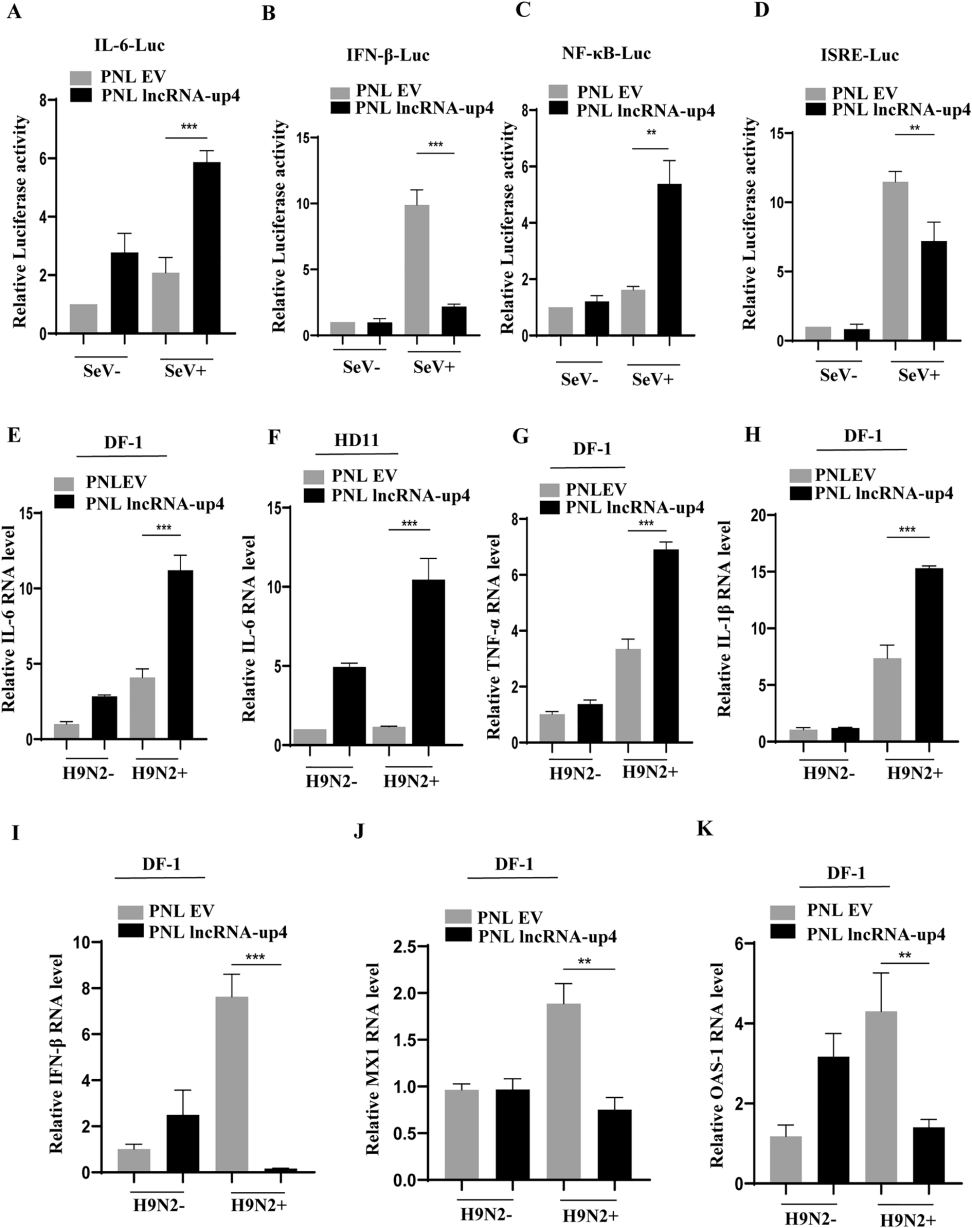
**Overexpression of lncRNA-up4 impairs the antiviral response**.** A–D** Luciferase reporter assays in lncRNA-up4-overexpressing cells. The data revealed increased IL-6 and NF-κB activation but suppressed IFN-β and ISRE responses associated with lncRNA-up4 overexpression. **E**, **F** qRT‒PCR confirmed elevated IL-6 mRNA levels in lncRNA-up4-overexpressing DF-1 and HD11 cells following H9N2 infection. **G**, **H** Overexpression of lncRNA-up4 significantly increased TNF-α and IL-1β mRNA expression in DF-1 cells after viral infection. **I–K** IFN-β, MX1, and OAS-1 mRNA levels were markedly lower in lncRNA-up4-overexpressing cells than in control cells after H9N2 infection. All luciferase assays and qRT‒PCR data are representative of three independent experiments with consistent results. The data are presented as the mean ± SD. ^**^p < 0.01, ^***^p < 0.001.

To confirm the findings from the lncRNA-up4 knockdown studies, the effects of lncRNA-up4 overexpression on the antiviral response were analysed. The results revealed that lncRNA-up4 overexpression increased IL-6 expression and NF-κB activation but suppressed IFN-β and ISRE responses, supporting its role in promoting inflammation while dampening IFN-mediated antiviral immunity (Figures 7A–D).

To further validate the role of lncRNA-up4 in regulating cytokine and ISG expression, lncRNA-up4 was overexpressed in DF-1 and HD11 cells, followed by H9N2 infection. Indeed, we observed that lncRNA-up4 significantly upregulated the production of proinflammatory cytokines, including IL-6, TNF-α, and IL-1β (Figures 7E–H), but significantly downregulated the expression of IFN-β, MX1 and OAS-1 (Figures 7I–K). Taken together, these results suggest that lncRNA-up4 is involved in regulating the expression of some cytokines and ISGs, potentially impacting innate immune responses and thereby affecting viral replication.

## Discussion

IAVs are highly transmissible viruses that cause respiratory disorders in humans and animals [40]. Although H9N2 is the type of LPAIV that causes respiratory disease but has mild clinical signs in chickens [41], it is critical for the adaptive evolution and interspecies transmission of the influenza virus. Viral infections lead to substantial transcriptional changes, including the transcription of lncRNAs from both host and viral genomes [42]. The functions of lncRNAs have been extensively studied in various species, specifically in humans. However, their roles in chickens are poorly understood. In particular, little information is available concerning the functional involvement of chicken lncRNAs in host–virus interactions [34, 43]. Recently, a few lncRNAs, such as MSTRG.14220.1, MSTRG.25416.43, MSTRG.24743.3, MSTRG.6458.31, and MSTRG.21445.2, were shown to play a role in IBV infection by regulating innate immune response genes in chicken HD11 cells [34]. Moreover, three chicken lncRNAs, XLOC-672329, XLOC-016500, and ALDBGALG0000001429, are thought to be involved in the host antiviral response during avian leukosis virus subgroup J (ALV-J) infection [44]. Despite their importance, a substantial number of chicken lncRNAs remain to be investigated.

In this study, we identified lncRNA-up4 as a novel immune-associated chicken lncRNA. It was significantly upregulated in DF-1 and HD11 chicken cells following IAV infection. Homology analysis revealed that lncRNA-up4 is absent in mammals (human/mouse) but is partially conserved in avian species (*Gallus gallus*, *Anser cygnoides*, *Anas platyrhynchos*, and *Falco* spp.), suggesting that potential functional analogues may exist in birds. Our findings demonstrated that during H9N2 AIV infection, lncRNA-up4 was significantly upregulated in multiple tissues, with the highest expression in the lungs and trachea. This tissue-specific expression pattern correlated with active viral replication, as supported by the increased expression of viral NP in these tissues. Notably, the upregulation of lncRNA-up4 in the liver and spleen upon IAV infection may suggest its potential role in the systemic immune response of the host, which is possibly mediated by circulating cytokines induced by viral infection. This behaviour mirrors that of other IAV-responsive lncRNAs, such as NEAT1, lncRNA-155, ISR, and IVPRIE [29]. Furthermore, functional analysis revealed that lncRNA-up4 acted as a negative regulator of the IFN-mediated antiviral response but positively regulated the expression of several proinflammatory cytokines and viral replication. In addition, subcellular fractionation analysis revealed that lncRNA-up4 was localized in both the nucleus and the cytoplasm but was more concentrated in the nucleus and less concentrated in the cytoplasm. These findings suggest that lncRNA-up4 may function in regulating gene expression at the transcriptional level, either by modulating chromatin accessibility, interacting with transcription factors, or acting as a molecular scaffold for gene regulation [45, 46]. The fact that some lncRNA-up4 was also localized in the cytoplasm may indicate that it is potentially functionally important; for example, it may act as a competing endogenous RNA (ceRNA) to influence mRNA stability inside the cytoplasm [47]. However, the precise molecular mechanisms underlying the relationship between lncRNA-up4 localization and function remain to be investigated.

We found that stimulation with poly(I:C), a synthetic analogue of double-stranded viral RNA, induced lncRNA-up4 expression in a dose-dependent manner, suggesting that it is involved in PRR-mediated antiviral signalling. In addition, this study provided mechanistic insights into how IAV may manipulate host immune signalling to induce lncRNA-up4 expression. TLR3 and MDA5 recognize viral dsRNA, activating downstream NF-κB p65 and IRF7 pathways to drive an immune response [48]. LPS, a PAMP, activates TLRs, triggering downstream signalling cascades such as the NF-κB pathway, which drive proinflammatory cytokine production [49]. The robust induction of lncRNA-up4 suggests its association with the pathway. The inhibition of NF-κB expression significantly reduced IAV-induced lncRNA-up4 expression, confirming that lncRNA-up4 was dependent on NF-κB. These findings align with those of previous studies showing that some lncRNAs are regulated by PRR signalling pathways and can act as regulatory elements in these pathways. For example, lncRNA-155 and lncRNA-NEAT1 have been shown to modulate NF-κB- and IRF7-dependent antiviral responses in mammalian systems [50, 51]. Innate immune sensors, particularly PRRs such as TLR3, MDA5, and RIG-I, are crucial for detecting viral RNA and triggering antiviral responses [52]. Our data provide evidence that chicken lncRNA-up4 may be involved in the NF-κB-dependent immune response.

LncRNAs regulate the expression of neighbouring genes through chromatin remodelling and modulation at multiple levels, including the transcriptional, post-transcriptional, translational, and post-translational levels [53]. The genomic location of lncRNA-up4 upstream of the IL-6 gene in chickens may imply its involvement in the regulation of the inflammatory response. Some lncRNAs located upstream of known protein-coding genes may act as enhancers to regulate neighbouring genes [54, 55]. Indeed, functional analysis revealed that knockdown and overexpression of lncRNA-up4 affected the expression of several key inflammatory cytokines, including IL-6, TNF-α, and IL-1β. Knockdown of lncRNA-up4 following H9N2 AIV infection significantly reduced the expression of these proinflammatory cytokines, whereas overexpression of lncRNA-up4 increased their expression. Because IL-6 and TNF-α are major drivers of inflammation, these results suggest that lncRNA-up4 may regulate viral replication through altering inflammatory responses during IAV infection. It has been shown that lncRNA-NEAT1 is a proinflammatory regulator that increases the expression of IL-6, IL-8, IL-1β, and TNF-α [56]. It has been suggested that the interplay of lncRNAs with TNF-α and IL-6, both pivotal mediators of inflammation, underscores their shared involvement in the inflammatory response [57]. Similarly, the binding of the lncRNA PVT1 to TNF-α modulates the inflammatory response [58]. It has also been shown that the lncRNA Lethe negatively regulates NF-κB activation during viral infection [59]. Our experiments demonstrated that lncRNA-up4 enhanced the NF-κB-driven inflammatory response. However, the precise mechanism remains to be determined.

Moreover, this study revealed a role for lncRNA-up4 in regulating IFN-mediated antiviral responses. Knockdown of lncRNA-up4 led to a significant increase in IFN-β, MX1, and OAS-1 expression induced by IAV, while its overexpression suppressed such responses. These results suggest that lncRNA-up4 acts as a negative regulator of IFN signalling, aiding viral immune evasion. Similar regulatory effects have been reported for several lncRNAs, including lncRNA NRAV [31] and lncRNA TSPOAP1 [47], both of which suppress ISG expression and impair host antiviral immunity. The upregulation of inflammatory cytokines combined with interferon suppression may contribute to hyperinflammatory conditions such as cytokine storms, which can lead to life-threatening complications [60]. LncRNA-up4 appeared to suppress the interferon response while promoting inflammatory cytokine production. This finding is consistent with the findings of previous studies showing that some lncRNAs act as negative regulators of IFN signalling to facilitate viral replication [61]. Additionally, the dual effects of lncRNA-up4 suppressing IFN-β while promoting IL-6 and TNF-α expression may suggest a coordinated immune modulation strategy that facilitates viral immune evasion. However, how lncRNA-up4 is involved in regulating the expression of these molecules remains unclear. Given its primarily nuclear localization, lncRNA-up4 may function via chromatin remodelling or transcription factor interactions, as reported for lncRNAs such as NEAT1 and lincRNA-EPS [62, 63]. Further mechanistic studies are needed to clarify its role.

In summary, we identified lncRNA-up4 as a negative regulator of innate immunity that contributes to the suppression of the IFN response following AIV infection, thereby facilitating viral replication. Notably, altering lncRNA-up4 expression significantly affected the production of the proinflammatory cytokines IL-6, TNF-α, and IL-1β, suggesting its involvement in regulating the inflammatory response to viral infection. Collectively, these findings provide new insights into the roles of chicken lncRNAs in host–virus interactions. Although this study specifically highlights the function of lncRNA-up4 in H9N2 infection, whether it also acts to regulate other IAV subtypes or respiratory viruses remains to be determined. In addition, further investigations are needed to elucidate the precise molecular mechanisms underlying its involvement in antiviral innate immunity.

## Supplementary Information

 **Additional file 1. The sequences of the primers and shRNAs used in this study**.

**Additional file 2. Differentially expressed lncRNAs induced by IAV infection.** (**A**) PCA plot of RNA-seq data showing clustering of biological replicates and separation between control and H9N2-infectedsamples on the basis of gene expression profiles. (**B**) The results from the top 30 enriched terms from theGO analysis are illustrated in three different categories (biological, cellular, and molecular levels) inorder of the log10 p-value for each entry. (**C**) RT‒PCR analysis showing the expression of lncRNA-up2,lncRNA-up4, lncRNA-up11, and NP in DF-1 cells after H9N2 infection. (**D**–**G**) Time-course analysis byRT‒PCR and qRT‒PCR of lncRNA-up2, lncRNA-up4, and lncRNA-up11 expression in HD11 cellsinfected with WSN or PR8 at 0, 6, 8, and 12 h post-infection (hpi). (**H**) Subcellular localization oflncRNA-up4, U6, and GAPDH in uninfected and infected samples. The percentage indicates thedistribution across the cytoplasm and nucleus. The data from three independent experiments are shown.All RT‒PCR and qRT‒PCR data represent three independent experiments with similar results. The dataare presented as the mean ± SD. *p < 0.05, **p < 0.01, ***p < 0.001.

**Additional file 3. Virus-induced lncRNA-up4 expression is regulated by PRR-dependent innateimmune signalling.** (**A**) Poly(I:C) stimulation led to dose-dependent upregulation of lncRNA-up4expression, as validated by RT‒PCR. MX1 expression confirmed the activation of the antiviral responseby poly(I:C) treatment. (**B**–**C**) TLR3 and MDA5 knockdown impaired virus-induced lncRNA-up4expression, as determined by RT‒PCR. All RT‒PCR experiments are representative of three independentexperiments with similar results.

**Additional file 4. NF-κB-mediated signalling plays a key role in the regulation of virus-inducedlncRNA-up4 expression.** (**A**) Dose-dependent increases in lncRNA-up4 and IL-6 mRNA expressionwere observed in DF-1 cells treated with LPS for 6 h, as validated by RT‒PCR. (**B**) RT‒PCR analysis oflncRNA-up4 expression in HD11 cells treated with Bay 11-7082 or the DMSO control and infected withH9N2 or mock-infected. (**C**) Transduction efficiency is indicated by GFP positivity from the vectorexpressing shRNAs. (**D**) RT‒PCR analysis of lncRNA-up4, NP and NF-κB p65 expression in DF-1 cellsfollowing NF-κB p65 knockdown and infection with H9N2 or mock-infection. (**E**) Transductionefficiency is indicated by GFP positivity as described in (C). (**F**) RT‒PCR analysis of lncRNA-up4, NPand IRF7 expression in DF-1 cells following IRF7 knockdown and infection with H9N2 or mock-infection. All RT‒PCR experiments are representative of three independent experiments with similarresults..

**Additional file 5. Silencing lncRNA-up4 expression significantly impairs IAV replication.** (**A**-**C**) RT‒PCR and western blotting were performed to examine the effects of shRNA-mediated lncRNA-up4knockdown on viral replication in DF-1 cells following H9N2 (A), WSN (B), or PR8 (C) infection. Viralreplication is indicated by viral NP mRNA and protein levels. (**D**) HD11 cells were used in experimentsthat were performed as described in (A). (**E**) shRNA-mediated lncRNA-up4 knockdown was examinedin HD11 cells by qRT‒PCR. (**F**) The effect of lncRNA-up4 knockdown on viral NP mRNA expressionwas examined in HD11 cells by qRT‒PCR. The data are shown as the mean ± SD of three independentexperiments. **p < 0.01.

**Additional file 6. LncRNA-up4 overexpression increases IAV replication**. (**A**–**C**) DF-1 cells weretransduced with a lncRNA-up4 overexpression plasmid. At 12 hpi with H9N2, WSN and PR8 IAVs, theoverexpression efficiency of lncRNA-up4 and viral NP expression was determined by RT‒PCR andwestern blotting. (**D**–**E**) The expression of lncRNA-up4, viral NP mRNA and protein was examined byRT‒PCR and western blotting (D) or qRT‒PCR (F). The results revealed significant upregulation of viralNP mRNA and protein expression in HD11 cells overexpressing lncRNA-up4 after H9N2 infection. Theresults are presented as the means ± SD of three independent experiments. ***p < 0.001.

## Data Availability

All the data generated or analysed in this study are available in this published article and its supplementary information files.

## References

[1] Zhang Y, Chi X, Hu J, Wang S, Zhao S, Mao Y, Peng B, Chen J-L, Wang S (2023) LncRNA LINC02574 inhibits influenza A virus replication by positively regulating the innate immune response. Int J Mol Sci 24:7248 37108410 10.3390/ijms24087248 PMC10138361

[2] Shao W, Li X, Goraya MU, Wang S, Chen J-L (2017) Evolution of influenza a virus by mutation and re-assortment. Int J Mol Sci 18:1650 28783091 10.3390/ijms18081650 PMC5578040

[3] Islam A, Islam S, Flora MS, Amin E, Woodard K, Webb A, Webster RG, Webby RJ, Ducatez MF, Hassan MM (2023) Epidemiology and molecular characterization of avian influenza A viruses H5N1 and H3N8 subtypes in poultry farms and live bird markets in Bangladesh. Sci Rep 13:7912 37193732 10.1038/s41598-023-33814-8 PMC10188517

[4] He Z, Wang X, Lin Y, Feng S, Huang X, Zhao L, Zhang J, Ding Y, Li W, Yuan R (2023) Genetic characteristics of waterfowl-origin H5N6 highly pathogenic avian influenza viruses and their pathogenesis in ducks and chickens. Front Microbiol 14:1211355 37405154 10.3389/fmicb.2023.1211355 PMC10315182

[5] Bröjer C, Ågren EO, Uhlhorn H, Bernodt K, Mörner T, Désirée JS, Mattsson R, Zohari S, Thorén P (2009) Pathology of natural highly pathogenic avian influenza H5N1 infection in wild tufted ducks (*Aythya fuligula*). J Vet Diagn Invest 21:579–587 19737752 10.1177/104063870902100501

[6] Umar S, Guerin JL, Ducatez MF (2017) Low pathogenic avian influenza and coinfecting pathogens: a review of experimental infections in avian models. Avian Dis 61:3–15 28301244 10.1637/11514-101316-Review

[7] Dhama K, Chauhan RS, Kataria JM, Mahendran M, Tomar S (2005) Avian influenza: the current perspectives. J Immunol Immunopathol 7:1–33

[8] Raj S, Astill J, Alqazlan N, Boodhoo N, Hodgins DC, Nagy É, Mubareka S, Karimi K, Sharif S (2022) Transmission of H9N2 low pathogenicity avian influenza virus (LPAIV) in a challenge-transmission model. Vaccines 10:1040 35891204 10.3390/vaccines10071040 PMC9316524

[9] Cui H, Che G, de Jong MCM, Li X, Liu Q, Yang J, Teng Q, Li Z, Beerens N (2022) The PB1 gene from H9N2 avian influenza virus showed high compatibility and increased mutation rate after reassorting with a human H1N1 influenza virus. Virol J 19:20 35078489 10.1186/s12985-022-01745-x PMC8788113

[10] Gu M, Chen H, Li Q, Huang J, Zhao M, Gu X, Jiang K, Wang X, Peng D, Liu X (2014) Enzootic genotype S of H9N2 avian influenza viruses donates internal genes to emerging zoonotic influenza viruses in China. Vet Microbiol 174:309–315 25457363 10.1016/j.vetmic.2014.09.029

[11] Qi W, Jia W, Liu D, Li J, Bi Y, Xie S, Li B, Hu T, Du Y, Xing L (2018) Emergence and adaptation of a novel highly pathogenic H7N9 influenza virus in birds and humans from a 2013 human-infecting low-pathogenic ancestor. J Virol 92:e00921–17 29070694 10.1128/JVI.00921-17 PMC5752946

[12] Liu M, Li X, Yuan H, Zhou J, Wu J, Bo H, Xia W, Xiong Y, Yang L, Gao R (2015) Genetic diversity of avian influenza A (H10N8) virus in live poultry markets and its association with human infections in China. Sci Rep 5:7632 25591167 10.1038/srep07632 PMC5379002

[13] Nang NT, Lee JS, Song BM, Kang YM, Kim HS, Seo SH (2011) Induction of inflammatory cytokines and toll-like receptors in chickens infected with avian H9N2 influenza virus. Vet Res 42:64 21592354 10.1186/1297-9716-42-64 PMC3114738

[14] Chothe SK, Nissly RH, Lim L, Bhushan G, Bird I, Radzio-Basu J, Jayarao BM, Kuchipudi SV (2020) NLRC5 serves as a pro-viral factor during influenza virus infection in chicken macrophages. Front Cell Infect Microbiol 10:230 32509599 10.3389/fcimb.2020.00230 PMC7248199

[15] Liniger M, Summerfield A, Zimmer G, McCullough KC, Ruggli N (2012) Chicken cells sense influenza A virus infection through MDA5 and CARDIF signaling involving LGP2. J Virol 86:705–717 22072756 10.1128/JVI.00742-11 PMC3255855

[16] Sun Y, Jiang J, Tien P, Liu W, Li J (2018) IFN-λ: a new spotlight in innate immunity against influenza virus infection. Protein Cell 9:832–837 29332267 10.1007/s13238-017-0503-6 PMC6160391

[17] McNab F, Mayer-Barber K, Sher A, Wack A, O’Garra A (2015) Type I interferons in infectious disease. Nat Rev Immunol 15:87–103 25614319 10.1038/nri3787 PMC7162685

[18] Dussurget O, Bierne H, Cossart P (2014) The bacterial pathogen *Listeria monocytogenes* and the interferon family: type I, type II and type III interferons. Front Cell Infect Microbiol 4:50 24809023 10.3389/fcimb.2014.00050 PMC4009421

[19] Wells AI, Coyne CB (2018) Type III interferons in antiviral defenses at barrier surfaces. Trends Immunol 39:848–858 30219309 10.1016/j.it.2018.08.008 PMC6179363

[20] Gewaid H, Bowie AG (2024) Regulation of type I and type III interferon induction in response to pathogen sensing. Curr Opin Immunol 87:102424 38761566 10.1016/j.coi.2024.102424

[21] Mattick JS, Amaral PP, Carninci P, Carpenter S, Chang HY, Chen L-L, Chen R, Dean C, Dinger ME, Fitzgerald KA (2023) Long non-coding RNAs: definitions, functions, challenges and recommendations. Nat Rev Mol Cell Biol 24:430–447 36596869 10.1038/s41580-022-00566-8 PMC10213152

[22] Yaghoobi A, Rezaee M, Behnoush AH, Khalaji A, Mafi A, Houjaghan AK, Masoudkabir F, Pahlavan S (2024) Role of long noncoding RNAs in pathological cardiac remodeling after myocardial infarction: an emerging insight into molecular mechanisms and therapeutic potential. Biomed Pharmacother 172:116248 38325262 10.1016/j.biopha.2024.116248

[23] Segal D, Dostie J (2023) The talented lncRNAs: meshing into transcriptional regulatory networks in cancer. Cancers (Basel) 15:3433 37444543 10.3390/cancers15133433 PMC10340227

[24] Kumar P, Bhandari N (2022) lncRNAs: Role in Regulation of Gene Expression. In: Uchiumi F (eds) Gene Expression, Chapter 4. Intech Open, London

[25] Verma S, Sahu BD, Mugale MN (2023) Role of lncRNAs in hepatocellular carcinoma. Life Sci 325:121751 37169145 10.1016/j.lfs.2023.121751

[26] Liu SJ, Dang HX, Lim DA, Feng FY, Maher CA (2021) Long noncoding RNAs in cancer metastasis. Nat Rev Cancer 21:446–460 33953369 10.1038/s41568-021-00353-1 PMC8288800

[27] Wu G-C, Pan H-F, Leng R-X, Wang D-G, Li X-P, Li X-M, Ye D-Q (2015) Emerging role of long noncoding RNAs in autoimmune diseases. Autoimmun Rev 14:798–805 25989481 10.1016/j.autrev.2015.05.004

[28] Kesheh MM, Mahmoudvand S, Shokri S (2022) Long noncoding RNAs in respiratory viruses: a review. Rev Med Virol 32:e2275 34252234 10.1002/rmv.2275 PMC8420315

[29] Wang J, Cen S (2020) Roles of lncRNAs in influenza virus infection. Emerg Microbes Infect 9:1407–1414 32543285 10.1080/22221751.2020.1778429 PMC7473136

[30] Ouyang J, Hu J, Chen J-L (2016) LncRNAs regulate the innate immune response to viral infection. Wiley Interdiscip Rev RNA 7:129–143 26667656 10.1002/wrna.1321 PMC7169827

[31] Chi X, Huang G, Wang L, Zhang X, Liu J, Yin Z, Guo G, Chen Y, Wang S, Chen J-L (2024) A small protein encoded by PCBP1-AS1 is identified as a key regulator of influenza virus replication via enhancing autophagy. PLoS Pathog 20:e1012461 39137200 10.1371/journal.ppat.1012461 PMC11343454

[32] Ouyang J, Zhu X, Chen Y, Wei H, Chen Q, Chi X, Qi B, Zhang L, Zhao Y, Gao GF (2014) NRAV, a long noncoding RNA, modulates antiviral responses through suppression of interferon-stimulated gene transcription. Cell Host Microbe 16:616–626 25525793 10.1016/j.chom.2014.10.001 PMC7104942

[33] Maarouf M, Chen B, Chen Y, Wang X, Rai KR, Zhao Z, Liu S, Li Y, Xiao M, Chen J-L (2019) Identification of lncRNA-155 encoded by MIR155HG as a novel regulator of innate immunity against influenza A virus infection. Cell Microbiol 21:e13036 31045320 10.1111/cmi.13036

[34] Li H, Cui P, Fu X, Zhang L, Yan W, Zhai Y, Lei C, Wang H, Yang X (2021) Identification and analysis of long non-coding RNAs and mRNAs in chicken macrophages infected with avian infectious bronchitis coronavirus. BMC Genomics 22:67 33472590 10.1186/s12864-020-07359-3 PMC7816148

[35] He Y, Ding Y, Zhan F, Zhang H, Han B, Hu G, Zhao K, Yang N, Yu Y, Mao L (2015) The conservation and signatures of lincRNAs in Marek’s disease of chicken. Sci Rep 5:15184 26471251 10.1038/srep15184 PMC4608010

[36] Han B, He Y, Zhang L, Ding Y, Lian L, Zhao C, Song J, Yang N (2017) Long intergenic non-coding RNA GALMD3 in chicken Marek’s disease. Sci Rep 7:10294 28860661 10.1038/s41598-017-10900-2 PMC5579197

[37] Wang S, Li H, Chen Y, Wei H, Gao GF, Liu H, Huang S, Chen J-L (2012) Transport of influenza virus neuraminidase (NA) to host cell surface is regulated by ARHGAP21 and Cdc42 proteins. J Biol Chem 287:9804–9816 22318733 10.1074/jbc.M111.312959 PMC3323004

[38] Mifsud EJ, Kuba M, Barr IG (2021) Innate immune responses to influenza virus infections in the upper respiratory tract. Viruses 13:2090 34696520 10.3390/v13102090 PMC8541359

[39] Hong J, Chi X, Yuan X, Wen F, Rai KR, Wu L, Song Z, Wang S, Guo G, Chen J-L (2022) I226r protein of African swine fever virus is a suppressor of innate antiviral responses. Viruses 14:575 35336982 10.3390/v14030575 PMC8951476

[40] Meseko C, Sanicas M, Asha K, Sulaiman L, Kumar B (2023) Antiviral options and therapeutics against influenza: history, latest developments and future prospects. Front Cell Infect Microbiol 13:1269344 38094741 10.3389/fcimb.2023.1269344 PMC10716471

[41] Wang S, Jiang N, Shi W, Yin H, Chi X, Xie Y, Hu J, Zhang Y, Li H, Chen J-L (2021) Co-infection of H9N2 influenza A virus and *Escherichia coli* in a BALB/c mouse model aggravates lung injury by synergistic effects. Front Microbiol 12:670688 33968006 10.3389/fmicb.2021.670688 PMC8097157

[42] Wang P (2019) The opening of pandora’s box: an emerging role of long noncoding RNA in viral infections. Front Immunol 9:3138 30740112 10.3389/fimmu.2018.03138 PMC6355698

[43] You Z, Zhang Q, Liu C, Song J, Yang N, Lian L (2019) Integrated analysis of lncRNA and mRNA repertoires in Marek’s disease infected spleens identifies genes relevant to resistance. BMC Genomics 20:245 30922224 10.1186/s12864-019-5625-1 PMC6438004

[44] Dai M, Feng M, Xie T, Zhang X (2019) Long non-coding RNA and MicroRNA profiling provides comprehensive insight into non-coding RNA involved host immune responses in ALV-J-infected chicken primary macrophage. Dev Comp Immunol 100:103414 31200006 10.1016/j.dci.2019.103414

[45] Chen L-L (2016) Linking long noncoding RNA localization and function. Trends Biochem Sci 41:761–772 27499234 10.1016/j.tibs.2016.07.003

[46] Rashid F, Shah A, Shan G (2016) Long non-coding RNAs in the cytoplasm. Genomics Proteomics Bioinform 14:73–80 10.1016/j.gpb.2016.03.005 PMC4880952 27163185

[47] Wang Q, Zhang D, Feng W, Guo Y, Sun X, Zhang M, Guan Z, Duan M (2022) Long noncoding RNA TSPOAP1 antisense RNA 1 negatively modulates type I IFN signaling to facilitate influenza A virus replication. J Med Virol 94:557–566 30968963 10.1002/jmv.25483

[48] Karpala AJ, Bagnaud-Baule A, Goossens KE, Lowenthal JW, Bean AGD (2012) Ontogeny of the interferon system in chickens. J Reprod Immunol 94:169–174 22472789 10.1016/j.jri.2012.02.008

[49] Yan B, Yu X, Cai X, Huang X, Xie B, Lian D, Chen J, Li W, Lin Y, Ye J (2024) A review: the significance of toll-like receptors 2 and 4, and NF-κB signaling in endothelial cells during atherosclerosis. Front Biosci Landmark 29:161 10.31083/j.fbl2904161 38682207

[50] Liu B, Sun L, Liu Q, Gong C, Yao Y, Lv X, Lin L, Yao H, Su F, Li D (2015) A cytoplasmic NF-κB interacting long noncoding RNA blocks IκB phosphorylation and suppresses breast cancer metastasis. Cancer Cell 27:370–381 25759022 10.1016/j.ccell.2015.02.004

[51] Zhang Q, Chen C-Y, Yedavalli VSRK, Jeang K-T (2013) NEAT1 long noncoding RNA and paraspeckle bodies modulate HIV-1 posttranscriptional expression. mBio 4:e00596–12 23362321 10.1128/mBio.00596-12 PMC3560530

[52] Rehwinkel J, Gack MU (2020) RIG-I-like receptors: their regulation and roles in RNA sensing. Nat Rev Immunol 20:537–551 32203325 10.1038/s41577-020-0288-3 PMC7094958

[53] Villegas VE, Zaphiropoulos PG (2015) Neighboring gene regulation by antisense long non-coding RNAs. Int J Mol Sci 16:3251–3266 25654223 10.3390/ijms16023251 PMC4346893

[54] Faust T, Frankel A, D’Orso I (2012) Transcription control by long non-coding RNAs. Transcription 3:78–86 22414755 10.4161/trns.19349 PMC3337829

[55] Ørom UA, Derrien T, Beringer M, Gumireddy K, Gardini A, Bussotti G, Lai F, Zytnicki M, Notredame C, Huang Q (2010) Long noncoding RNAs with enhancer-like function in human cells. Cell 143:46–58 20887892 10.1016/j.cell.2010.09.001 PMC4108080

[56] Xia LX, Ke C, Lu JM (2018) NEAT1 contributes to neuropathic pain development through targeting miR-381/HMGB1 axis in CCI rat models. J Cell Physiol 233:7103–7111 29633273 10.1002/jcp.26526 PMC13482416

[57] Cestonaro LV, Macedo SMD, Piton YV, Garcia SC, Arbo MD (2022) Toxic effects of pesticides on cellular and humoral immunity: an overview. Immunopharmacol Immunotoxicol 44:816–831 35770924 10.1080/08923973.2022.2096466

[58] Zhang T, Yang Y-H, Liu Y-P, Zhang T-N, Yang N (2022) Regulatory role of noncoding RNA in sepsis and sepsis-associated organ dysfunction: an updated systematic review. Shock 58:434–456 36155389 10.1097/SHK.0000000000002000

[59] Xiong Y, Yuan J, Zhang C, Zhu Y, Kuang X, Lan L, Wang X (2015) The STAT3-regulated long non-coding RNA Lethe promote the HCV replication. Biomed Pharmacother 72:165–171 26054691 10.1016/j.biopha.2015.04.019

[60] Akhtar LN, Qin H, Muldowney MT, Yanagisawa LL, Kutsch O, Clements JE, Benveniste EN (2010) Suppressor of cytokine signaling 3 inhibits antiviral IFN-β signaling to enhance HIV-1 replication in macrophages. J Immunol 185:2393–2404 20631305 10.4049/jimmunol.0903563 PMC3935802

[61] Goraya MU, Zaighum F, Sajjad N, Anjum FR, Sakhawat I, ur Rahman S (2020) Web of interferon stimulated antiviral factors to control the influenza A viruses replication. Microb Pathog 139:103919 31830579 10.1016/j.micpath.2019.103919

[62] Imamura K, Imamachi N, Akizuki G, Kumakura M, Kawaguchi A, Nagata K, Kato A, Kawaguchi Y, Sato H, Yoneda M (2014) Long noncoding RNA NEAT1-dependent SFPQ relocation from promoter region to paraspeckle mediates IL8 expression upon immune stimuli. Mol Cell 53:393–406 24507715 10.1016/j.molcel.2014.01.009

[63] Atianand MK, Hu W, Satpathy AT, Shen Y, Ricci EP, Alvarez-Dominguez JR, Bhatta A, Schattgen SA, McGowan JD, Blin J (2016) A long noncoding RNA lincRNA-EPS acts as a transcriptional brake to restrain inflammation. Cell 165:1672–1685 27315481 10.1016/j.cell.2016.05.075 PMC5289747

