# Supplementary material for: A chicken lncRNA is identified as a critical regulator that increases influenza virus replication by impairing innate antiviral responses
Source: Vet Res. 2025 Oct 15;56:195. doi: 10.1186/s13567-025-01635-4 (PMC12523108; doi:10.1186/s13567-025-01635-4)
Supplement: Supplementary file 1 — Additional file 1. The sequences of the primers and shRNAs used in this study. [file 13567_2025_1635_MOESM1_ESM.pdf]

Additional file 1 The sequence of primers and shRNA used in this study

Gene	Sequence	Accession numbers
β-actin-F	5'AGACATCAGGGTGTGATGGTTGGT-3'	NM_205518.1
β-actin-R	5'TGGTGACAATACCGTGTTCAATGG-3'	
H9N2 NP-F	5'-TCAAACGTGGGATCAATG-3'	MT_774533
H9N2 NP-R	5'-GTGCAGACCGTGCTAAAA-3'	
lncRNA-up2-F	5'-AATCACAACCGTTCCCAATCC-3'	N/A
lncRNA-up2-R	5'-ACTACAGCTTCTTCTGTAATGGCT-3'	
lncRNA-up11-F	5'-CAGCTGATCTGTGATTGATTAT-3'	N/A
lncRNA-up11-R	5'-GCCACAACGGGGTGCACTGGA-3'	
lncRNA-up4-F	5'-CAAGTGCAAAGCGTGAGCAG-3'	N/A
lncRNA-up4-R	5'-ATGGGGACACCACCATTTCC-3'	
IL-6-F	5'-TTCGCCTTTCAGACCTACCTG-3'	NM_204628.1
IL-6-R	5'-ATAAGCGGCAGCGGGAAA-3'	
IL-1β-F	5'-CAGCAGCCTCAGCGAAGAG-3'	NM_204524.1
IL-1β-R	5'-CTGTGGTGTGCTCAGAATCCA-3'	
TNF-α-F	5'-GCCCTTCCTGTAACCAGATG-3'	NM_204267.1
TNF-α-R	5'-ACACGACAGCCAAGTCAACG-3'	
IFN-β-F	5'-CCTCAACCAGATCCAGCATT-3'	NM_001024836.1
IFN-β-R	5'-GGATGAGGCTGTGAGAGGAG-3'	
MX1-F	5'-AAGCCTGAGCATGAGCAGAA-3'	NM_204609.1
MX1-R	5'-TCTCAGGCTGTCAACAAGATCAA-3'	
OASI-F	5'-ACATCCTCGCCATCATCGA-3'	NM_204909.1
OASI-R	5'-GCGGACTGGTGATGCTGACT-3'	
IRF7-F	5'-AACGACGACCCGCACAAG-3'	NM_001007079.1
IRF7-R	5'-GCAGCAGGTCCAAATCCA-3'	
TLR3-F	5'-TCAGTACATTTGTAACACCCCGCC-3'	NM_001011691.2
TLR3-R	5'-GGCGTCATAATCAAACACTCC-3'	
NF-κB-F	5'-TCAACGCAGGACCTAAAGACAT-3'	NM_205129.1
NF-κB-R	5'-GCAGATAGCCAAGTTCAGGATG-3'	
MDA5-F	5'-TGAAAGCCTTGCAGATGACTTA-3'	NM_001012893.1
MDA5-R	5'-GCTGTTTCAAATCCTCCGTTAC-3'	
U6 nuclear-F	5'-GCGCGTCGTGGCGTGCCCTGC-3'	NR_131753.1

U6 nuclear-R	5’-GCGTTCTCGGCATGCTCTCGT-3’	
GAPDH-F	5’-GCCCAGAACATCATCCCA-3’	NM_204305.1
GAPDH-R	5’-CGGCAGGTCAGGTCAACA-3’	
sh-lncRNA-up4-F	5’-GCCATTCATGGCACAGCAACT-3’	N/A
sh-lncRNA-up4-R	5’-AGTTGCTGTGCCATGAATGGC-3’	
sh-IRF7-F	5’-GGAGCACTCACATGTTCATGC-3’	NM_001007079.1
sh-IRF7-R	5’-GCATGAACATGTGAGTGCTCC-3’	
sh-TLR3-F	5’-GCTTGGACATTTCCCATAATC-3’	NM_001011691.2
sh-TLR3-R	5’-GATTATGGGAAATGTCCAAGC-3’	
sh-NF-κB-F	5’-GCTGCTCTTCAGCAAGTTTCC-3’	NM_205129.1
sh-NF-κB-R	5’-GGAAACTTGCTGAAGAGCAGC-3’	
sh-MDA5-F	5’-GGTTGGGATTGAGACTGTTAT-3’	NM_001012893.1
sh-MDA5-R	5’-ATAACAGTCTCAATCCCAACC-3’	
