# Supplementary figures and images for: A chicken lncRNA is identified as a critical regulator that increases influenza virus replication by impairing innate antiviral responses
Source: Vet Res. 2025 Oct 15;56:195. doi: 10.1186/s13567-025-01635-4 (PMC12523108; doi:10.1186/s13567-025-01635-4)

Additional file 2

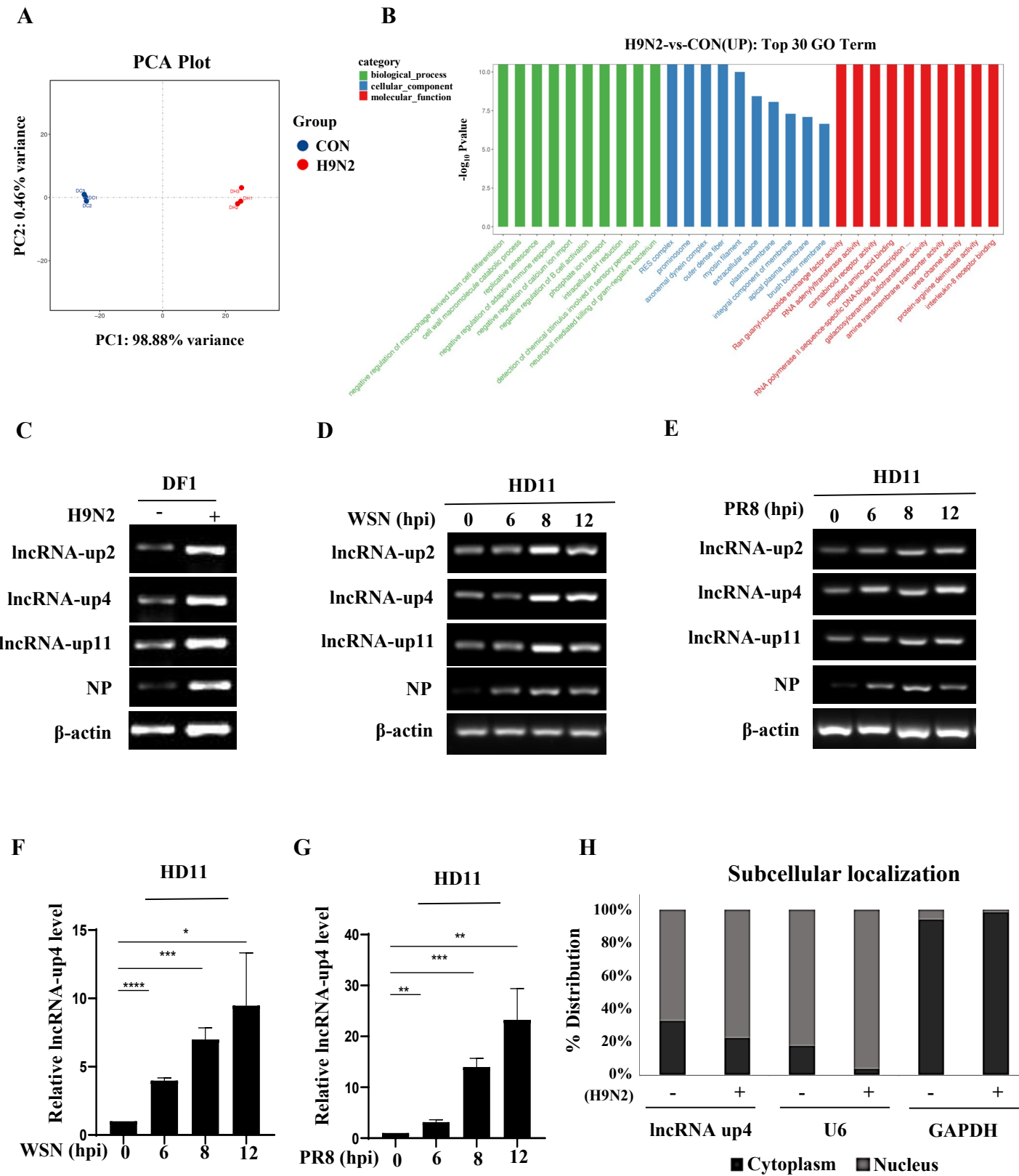

Supplement: Supplementary file 2 — Additional file 2. Differentially expressed lncRNAs induced by IAV infection. (A) PCA plot of RNA-seq data showing clustering of biological replicates and separation between control and H9N2-infectedsamples on the basis of gene expression profiles. (B) The results from the top 30 enriched terms from theGO analysis are illustrated in three different categories (biological, cellular, and molecular levels) inorder of the log10 p-value for each entry. (C) RT‒PCR analysis showing the expression of lncRNA-up2,lncRNA-up4, lncRNA-up11, and NP in DF-1 cells after H9N2 infection. (D–G) Time-course analysis byRT‒PCR and qRT‒PCR of lncRNA-up2, lncRNA-up4, and lncRNA-up11 expression in HD11 cellsinfected with WSN or PR8 at 0, 6, 8, and 12 h post-infection (hpi). (H) Subcellular localization oflncRNA-up4, U6, and GAPDH in uninfected and infected samples. The percentage indicates thedistribution across the cytoplasm and nucleus. The data from three independent experiments are shown.All RT‒PCR and qRT‒PCR data represent three independent experiments with similar results. The dataare presented as the mean ± SD. *p < 0.05, **p < 0.01, ***p < 0.001. [file 13567_2025_1635_MOESM2_ESM.pdf]

Additional file 3

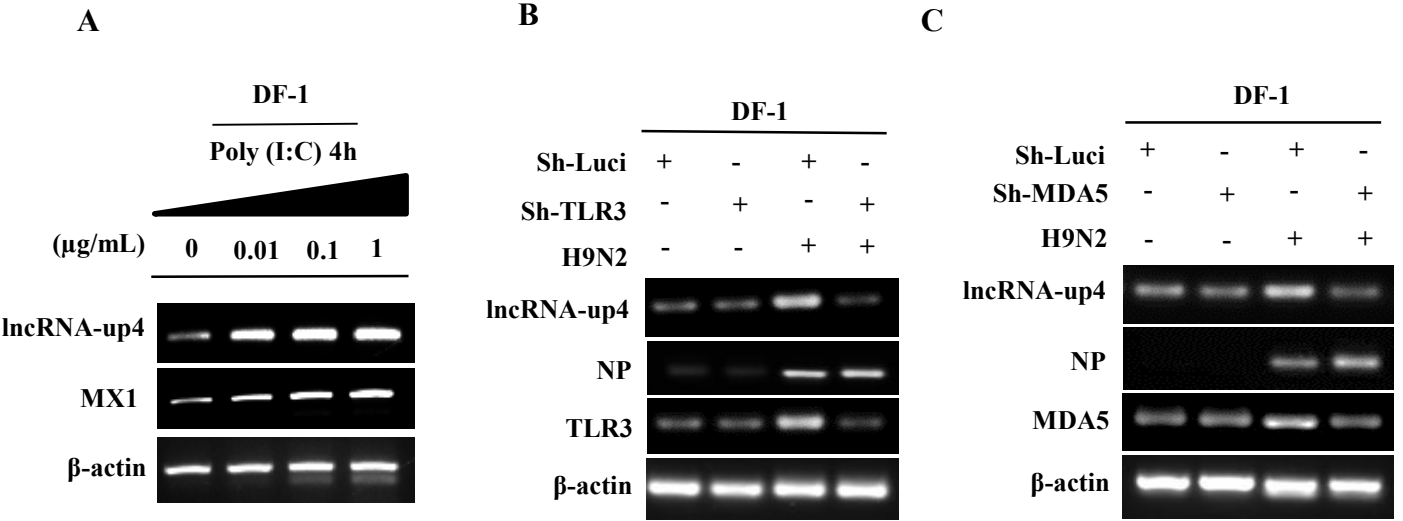

Supplement: Supplementary file 3 — Additional file 3. Virus-induced lncRNA-up4 expression is regulated by PRR-dependent innateimmune signalling. (A) Poly(I:C) stimulation led to dose-dependent upregulation of lncRNA-up4expression, as validated by RT‒PCR. MX1 expression confirmed the activation of the antiviral responseby poly(I:C) treatment. (B–C) TLR3 and MDA5 knockdown impaired virus-induced lncRNA-up4expression, as determined by RT‒PCR. All RT‒PCR experiments are representative of three independentexperiments with similar results. [file 13567_2025_1635_MOESM3_ESM.pdf]

Additional file 4

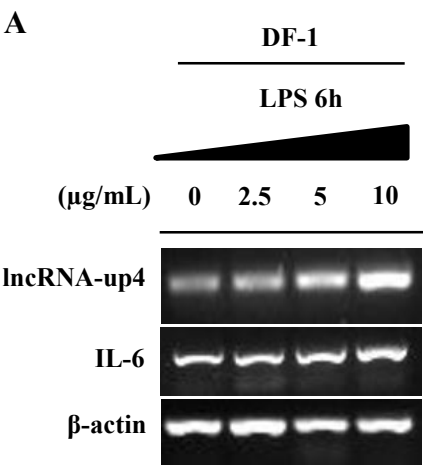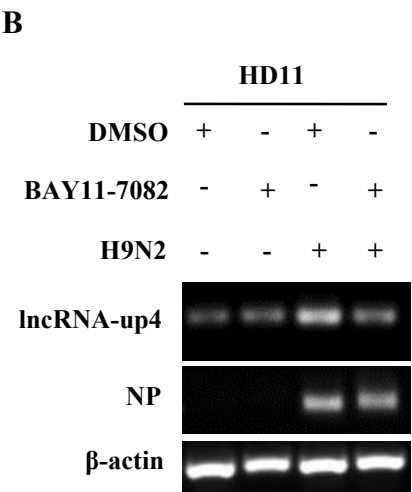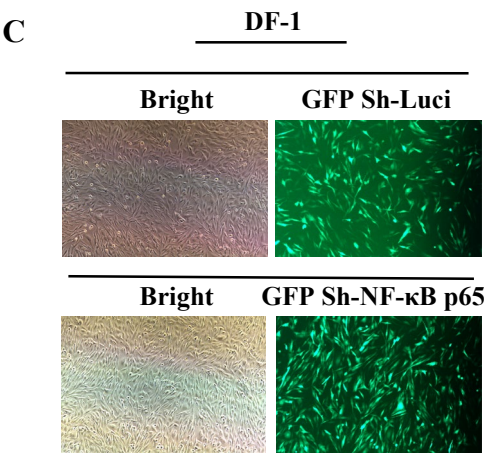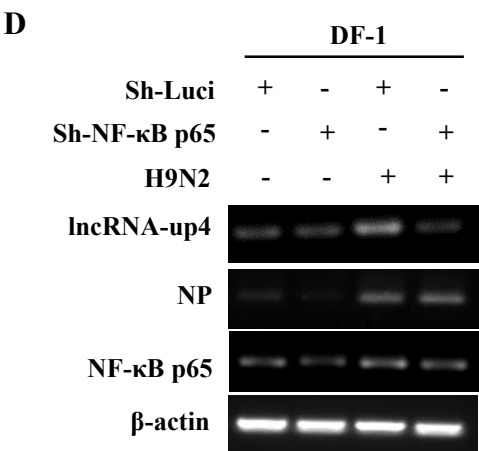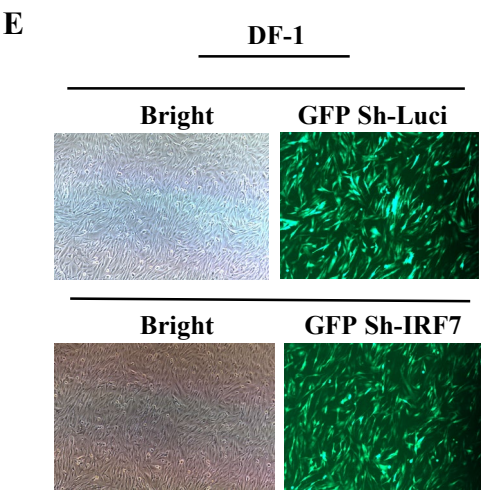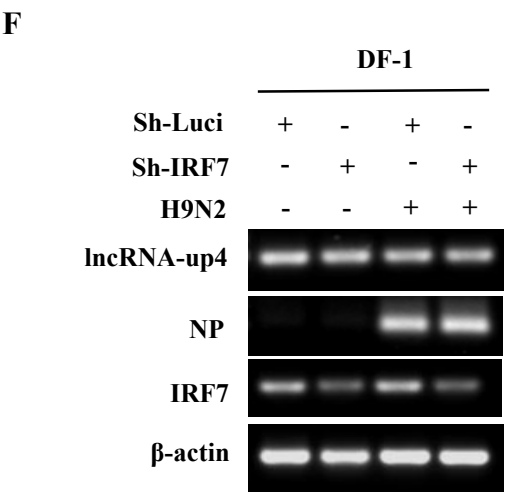

Supplement: Supplementary file 4 — Additional file 4. NF-κB-mediated signalling plays a key role in the regulation of virus-inducedlncRNA-up4 expression. (A) Dose-dependent increases in lncRNA-up4 and IL-6 mRNA expressionwere observed in DF-1 cells treated with LPS for 6 h, as validated by RT‒PCR. (B) RT‒PCR analysis oflncRNA-up4 expression in HD11 cells treated with Bay 11-7082 or the DMSO control and infected withH9N2 or mock-infected. (C) Transduction efficiency is indicated by GFP positivity from the vectorexpressing shRNAs. (D) RT‒PCR analysis of lncRNA-up4, NP and NF-κB p65 expression in DF-1 cellsfollowing NF-κB p65 knockdown and infection with H9N2 or mock-infection. (E) Transductionefficiency is indicated by GFP positivity as described in (C). (F) RT‒PCR analysis of lncRNA-up4, NPand IRF7 expression in DF-1 cells following IRF7 knockdown and infection with H9N2 or mock-infection. All RT‒PCR experiments are representative of three independent experiments with similarresults.. [file 13567_2025_1635_MOESM4_ESM.pdf]

Additional file 5

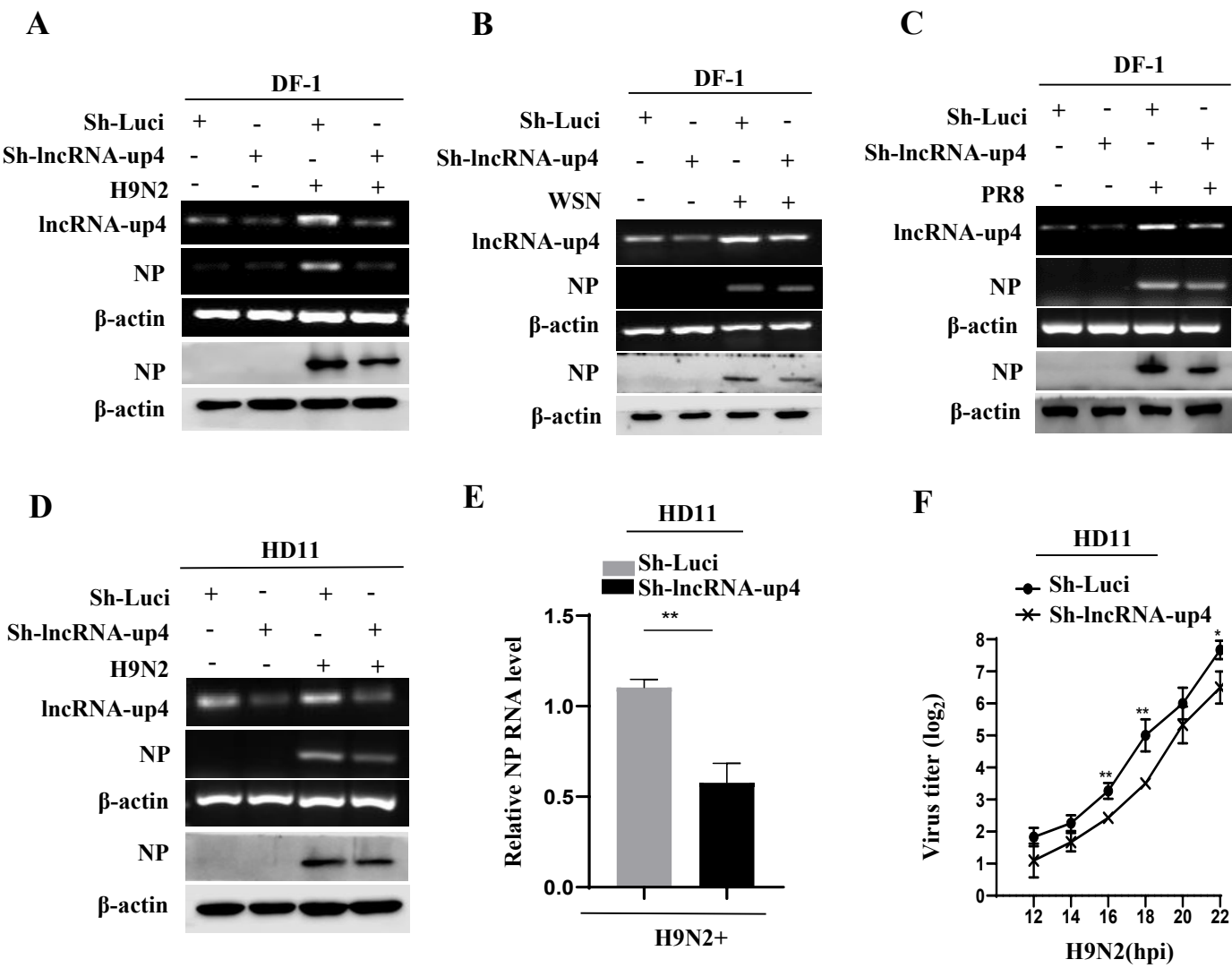

Supplement: Supplementary file 5 — Additional file 5. Silencing lncRNA-up4 expression significantly impairs IAV replication. (A-C) RT‒PCR and western blotting were performed to examine the effects of shRNA-mediated lncRNA-up4knockdown on viral replication in DF-1 cells following H9N2 (A), WSN (B), or PR8 (C) infection. Viralreplication is indicated by viral NP mRNA and protein levels. (D) HD11 cells were used in experimentsthat were performed as described in (A). (E) shRNA-mediated lncRNA-up4 knockdown was examinedin HD11 cells by qRT‒PCR. (F) The effect of lncRNA-up4 knockdown on viral NP mRNA expressionwas examined in HD11 cells by qRT‒PCR. The data are shown as the mean ± SD of three independentexperiments. **p < 0.01. [file 13567_2025_1635_MOESM5_ESM.pdf]

Additional file 6

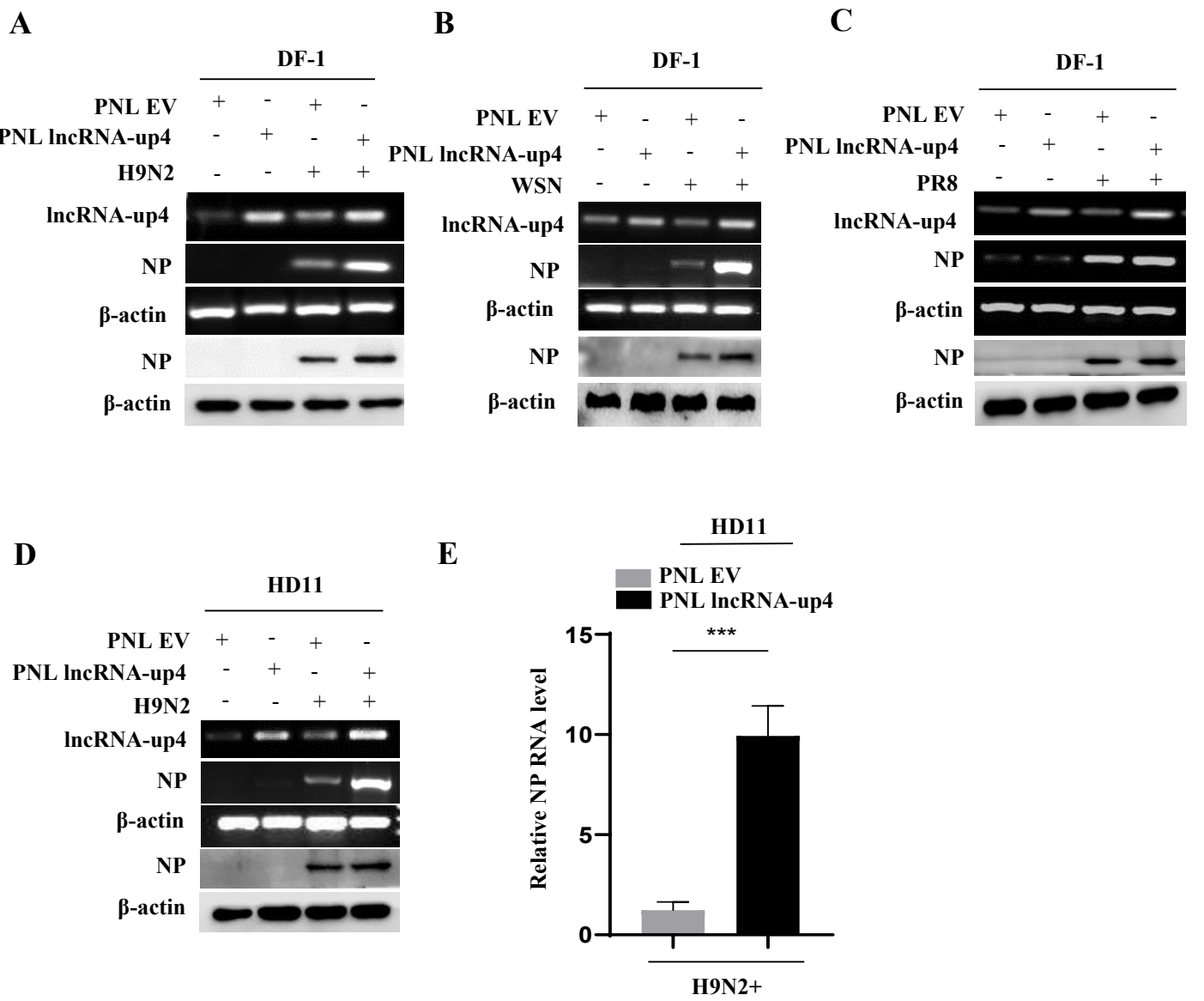

Supplement: Supplementary file 6 — Additional file 6. LncRNA-up4 overexpression increases IAV replication. (A–C) DF-1 cells weretransduced with a lncRNA-up4 overexpression plasmid. At 12 hpi with H9N2, WSN and PR8 IAVs, theoverexpression efficiency of lncRNA-up4 and viral NP expression was determined by RT‒PCR andwestern blotting. (D–E) The expression of lncRNA-up4, viral NP mRNA and protein was examined byRT‒PCR and western blotting (D) or qRT‒PCR (F). The results revealed significant upregulation of viralNP mRNA and protein expression in HD11 cells overexpressing lncRNA-up4 after H9N2 infection. Theresults are presented as the means ± SD of three independent experiments. ***p < 0.001. [file 13567_2025_1635_MOESM6_ESM.pdf]
